# Safety and immunogenicity of the oral, inactivated, enterotoxigenic *Escherichia coli* vaccine ETVAX in Bangladeshi children and infants: a double-blind, randomised, placebo-controlled phase 1/2 trial

**DOI:** 10.1016/S1473-3099(19)30571-7

**Published:** 2020-02

**Authors:** Firdausi Qadri, Marjahan Akhtar, Taufiqur R Bhuiyan, Mohiul I Chowdhury, Tasnuva Ahmed, Tanzeem A Rafique, Arifuzzaman Khan, Sadia I A Rahman, Farhana Khanam, Anna Lundgren, Gudrun Wiklund, Joanna Kaim, Madeleine Löfstrand, Nils Carlin, A Louis Bourgeois, Nicole Maier, Alan Fix, Thomas Wierzba, Richard I Walker, Ann-Mari Svennerholm

**Affiliations:** a International Centre for Diarrhoeal Disease Research Bangladesh, Dhaka, Bangladesh; b Gothenburg University Vaccine Research Institute, Department of Microbiology and Immunology, Institute of Biomedicine, University of Gothenburg, Gothenburg, Sweden; c Scandinavian Biopharma, Solna, Sweden; d PATH, Washington DC, USA; e Wake Forest School of Medicine, Section on Infectious Diseases, Department of Internal Medicine, Winston Salem, NC, USA

## Abstract

**Background:**

Enterotoxigenic *Escherichia coli* causes diarrhoea, leading to substantial mortality and morbidity in children, but no specific vaccine exists. This trial tested an oral, inactivated, enterotoxigenic *E coli* vaccine (ETVAX), which has been previously shown to be safe and highly immuongenic in Swedish and Bangladeshi adults. We tested the safety and immunogenicity of ETVAX, consisting of four *E coli* strains overexpressing the most prevalent colonisation factors (CFA/I, CS3, CS5, and CS6) and a toxoid (LCTBA) administered with or without a double-mutant heat-labile enterotoxin (dmLT) as an adjuvant, in Bangladeshi children.

**Methods:**

We did a randomised, double-blind, placebo-controlled, dose-escalation, age-descending, phase 1/2 trial in Dhaka, Bangladesh. Healthy children in one of three age groups (24–59 months, 12–23 months, and 6–11 months) were eligible. Children were randomly assigned with block randomisation to receive either ETVAX, with or without dmLT, or placebo. ETVAX (half [5·5 × 10^10^ cells], quarter [2·5 × 10^10^ cells], or eighth [1·25 × 10^10^ cells] adult dose), with or without dmLT adjuvant (2·5 μg, 5·0 μg, or 10·0 μg), or placebo were administered orally in two doses 2 weeks apart. Investigators and participants were masked to treatment allocation. The primary endpoint was safety and tolerability, assessed in all children who received at least one dose of vaccine. Antibody responses to vaccine antigens, defined as at least a two-times increase in antibody levels between baseline and post-immunisation, were assessed as secondary endpoints. This trial is registered with ClinicalTrials.gov, NCT02531802.

**Findings:**

Between Dec 7, 2015, and Jan 10, 2017, we screened 1500 children across the three age groups, of whom 430 were enrolled and randomly assigned to the different treatment groups (130 aged 24–59 months, 100 aged 12–23 months, and 200 aged 6–11 months). All participants received at least one dose of vaccine. No solicited adverse events occurred that were greater than moderate in severity, and most were mild. The most common solicited event was vomiting (ten [8%] of 130 patients aged 24–59 months, 13 [13%] of 100 aged 12–23 months, and 29 [15%] of 200 aged 6–11 months; mostly of mild severity), which appeared related to dose and age. The addition of dmLT did not modify the safety profile. Three serious adverse events occurred but they were not considered related to the study drug. Mucosal IgA antibody responses in lymphocyte secretions were detected against all primary vaccine antigens (CFA/I, CS3, CS5, CS6, and the LCTBA toxoid) in most participants in the two older age groups, whereas such responses to four of the five antigens were less frequent and of lower magnitude in infants aged 6–11 months than in older children. Faecal secretory IgA immune responses were recorded against all vaccine antigens in infants aged 6–11 months. 78 (56%) of 139 infants aged 6–11 months who were vaccinated developed mucosal responses against at least three of the vaccine antigens versus 14 (29%) of 49 of the infants given placebo. Addition of the adjuvant dmLT enhanced the magnitude, breadth, and kinetics (based on number of responders after the first dose of vaccine) of immune responses in infants.

**Interpretation:**

The encouraging safety and immunogenicity of ETVAX and benefit of dmLT adjuvant in young children support its further assessment for protective efficacy in children in enterotoxigenic *E coli*-endemic areas.

**Funding:**

PATH (Bill & Melinda Gates Foundation and the UK's Department for International Development), the Swedish Research Council, and The Swedish Foundation for Strategic Research.

## Introduction

Enterotoxigenic *Escherichia coli* remains one of the most common bacterial pathogens causing diarrhoea, leading to substantial mortality and morbidity in children in low-income and middle-income countries (LMICs), and vaccine development remains a WHO priority.^1^, ^2^, ^3^, ^4^, ^5^, ^6^ Enterotoxigenic *E coli* causes disease by colonising the small intestine by expressing different colonisation factors on the bacterial surface and subsequently releasing heat-labile or heat-stable enterotoxins.^7^, ^8^, ^9^, ^10^ Immune protection is most likely provided by secretory IgA antibodies against colonisation factors and heat-labile toxin, which are produced in the intestine.^1^, ^10^

**Research in context**

**Evidence before this study**

Infection with enterotoxigenic *Escherichia coli* is a major cause of diarrhoea and stunting in children and infants, yet no licensed vaccine against this pathogen exists. The University of Gothenburg has been working on enterotoxigenic *E coli* vaccine development for more than two decades. During this time, the University of Gothenburg team and other collaborators have periodically reviewed the literature on this subject and published the results in numerous reports in peer reviewed journals. Based on careful monitoring of the literature, we found two other oral vaccine candidates for enterotoxigenic *E coli* that are approaching clinical development, both based on attenuated *Shigella* as vectors, and one injectable vaccine based on fimbrial tip adhesins, which is in early clinical development. An oral, attenuated enterotoxigenic *E coli* vaccine was shown to protect humans against challenge with live enterotoxigenic *E coli* bacteria (H10407) when administered with a double-mutant heat-labile enterotoxin (dmLT) adjuvant, but this candidate is no longer in active development. A series of clinical studies by the University of Gothenburg team indicated that the inactivated whole-cell approach would be promising for enterotoxigenic *E coli*. These data were reviewed by a WHO panel, which recommended overexpression of antigens on the cells comprising the vaccine and inclusion of an adjuvant in the formulation. In response, the University of Gothenburg team and collaborators developed ETVAX, which provided higher expression of enterotoxigenic *E coli* antigens than an earlier version of the vaccine and was shown to be safe when given to adult Swedish volunteers in conjunction with dmLT adjuvant. Subsequently, the team observed similar safety results in adults in Bangladesh.

**Added value of this study**

This trial assessed for the first time the safety and immunogenicity of ETVAX in young children and infants and was the first analysis of dmLT adjuvant with a vaccine in this population in a low-income and middle-income country (LMIC). By using *E coli* bacteria engineered to overexpress vaccine antigens, we showed that infants and children can develop significant intestinal immune responses to the vaccine. Furthermore, these responses can be improved in infants aged 6–11 months by administering dmLT with the vaccine. Moreover, this study showed that occasional mild to moderate vomiting can be reduced without loss of immunogenicity by reducing the dose of vaccine.

**Implications of all the available evidence**

These data show that ETVAX can be used to induce mucosal immune responses to the vaccine antigens and that use of dmLT might improve these responses in infants in LMICs, which would be the logical target group for this vaccine and a group who have been difficult to effectively immunise with other oral vaccines. The information gained in this study not only markedly advances the further development of ETVAX but could also have important implications for more successful use of other oral vaccines, such as cholera, among infants and young children in LMICs.

On the basis of WHO recommendations,^11^ we have developed an oral enterotoxigenic *E coli* vaccine (ETVAX) containing recombinant *E coli* strains overexpressing the most prevalent enterotoxigenic *E coli* colonisation factors (ie, CFA/I, CS3, CS5, and CS6) at substantially higher concentrations than expressed by clinical enterotoxigenic *E coli* isolates^10^, ^12^, ^13^ and combined with a heat-labile-like toxoid LCTBA (a hybrid molecule of cholera toxin B subunit [CTB] and heat-labile toxin B subunit [LTB]).^14^ ETVAX might be more immunogenic when used in combination with a mucosal adjuvant, the double-mutant heat-labile toxin (dmLT).^13^, ^15^

When tested in Swedish adults, ETVAX, with or without dmLT, was safe and induced statistically significant faecal secretory IgA and IgA antibody in lymphocyte secretions (ALS) responses against all colonisation factors and LTB in most vaccinees.^16^ ETVAX also induced long-lasting immunological memory in Swedish adults.^17^ On the basis of these results, we designed a large, dose-escalation, age-descending phase 1/2 trial of ETVAX in Bangladesh.

First, we assessed the safety and immunogenicity of ETVAX in adults aged 18–45 years in Bangladesh.^18^ The results in adults indicated that ETVAX was safe and induced ALS responses against all five primary vaccine antigens in 100% of vaccinees and strong plasma immune responses in 60–100%.^18^ On the basis of the safety data in adults, we proceeded to assess the vaccine in children, with the primary objective to establish the largest tolerated dose of ETVAX, with or without dmLT. A secondary objective was to assess the potential of the vaccine to induce mucosal and systemic immune responses against the five primary vaccine antigens.

## Materials and methods

### Study design and participants

We did a randomised, double-blind, placebo-controlled, dose-escalation and descending-age, phase 1/2 trial at the International Centre for Diarrhoeal Disease Research, Bangladesh (icddr,b) study area in Mirpur, Dhaka, Bangladesh. The study was performed in accordance with the Declaration of Helsinki and approved by the Research Review and Ethical Review Committees of the International Centre for Diarrhoeal Disease Research, Bangladesh; the Western Institutional Review Board, USA; and the US Food and Drug Administration. Written informed consent via signature, thumbprint, or signature of a third-party impartial witness was obtained from the parents of participants.

Healthy children aged 6–59 months were screened 4–7 days before enrolment for eligibility based on medical history, clinical examination, and laboratory tests. Key exclusion criteria were known systemic disorders that might affect safety or conformance to the protocol, congenital abdominal disorders, diarrhoea within 7 days of vaccination, febrile illness within 48 h before vaccination, medically significant malnutrition (weight-for-height Z score less than −2·0), current use of immunosuppressive drugs, use of antimicrobial drugs within 14 days before enrolment, and stool samples positive for diarrhoeal pathogens (rotavirus, enterotoxigenic *E coli, Shigella, Vibrio cholerae*, or *Salmonella)* within 7 days of vaccination. Full eligibility criteria are listed in the protocol (appendix p 24).

### Randomisation and masking

Participants were randomly assigned in their age groups to receive ETVAX at eighth, quarter, half, or full adult doses, with or without dmLT, or placebo. The planned number and allocation of participants and doses of vaccine and dmLT are in the appendix (p 1). We aimed to enrol twice as many infants in the age group 6–11 months than in the other groups because this is the main target group for an ETEC vaccine in LMICs. Block randomisation was used, and block size depended on the sample size and vaccine-to-placebo ratio within each age group and cohort. Randomisation occurred on the day participants were to receive their first immunisation after confirmation of eligibility. Participants were assigned to a masked treatment provided by the statistical group at The Emmes Corporation (Rockville, MD, USA), and each participant's treatment assignment was entered into the electronic data system after administration had occurred. The unmasked study pharmacist (or designee) maintained the treatment code list in a secure place. Parents, participants, and all study staff were masked to treatment assignment.

### Procedures

Two doses of ETVAX (produced for Scandinavian Biopharma by Biovian (Turku, Finland; lot BX1003574) were given orally to study participants, with a 2-week interval between the first and second doses. The full adult dose of vaccine consists of 1 × 10^11^ inactivated *E coli* bacteria (strains ETEX 21–24) recombinantly induced to express high amounts of CFA/I, CS3, CS5, or CS6 antigens, mixed with 1 mg of LCTBA protein, a recombinantly produced LTB/CTB hybrid protein that induces strong neutralising antibody responses to LTB.^10^, ^13^, ^14^ Fractional (half [5·5 × 10^10^ cells], quarter [2·5 × 10^10^ cells], or eighth [1·25 × 10^10^ cells] adult doses) and full doses of vaccine were suspended in bicarbonate buffer (Recipharm, Stockholm, Sweden) in buffer volumes of 30 mL for children aged 24–59 months, 15 mL for children aged 12–23 months, and 10 mL for children aged 6–11 months. The suspension was then given alone or together with 2·5–10·0 μg dmLT (R192G/L211A, lot 1575; Walter Reed Army Institute of Research, Silver Spring, MD, USA).^15^ Placebo recipients received the same volumes of buffer as the volume of vaccine plus buffer received by vaccine recipients for each age group. Vaccine or placebo was prepared by an unmasked pharmacist. Participants were not allowed to eat, drink, or be breastfed 1 h before and after vaccination.

Parents or caretakers of study participants were given memory aids to record solicited signs and symptoms during the 5–7 days after immunisation. Solicited reactogenicity events assessed during the week after each dose included fever, vomiting, loose stools, diarrhoea, abdominal pain, and acute systemic allergic reaction. Adverse events were assessed by trained study staff at home visits each day for 5–7 days after each immunisation, and at clinic visits 7 days and 14 days after the first dose, 5 days after the second dose, and 28 days and 182 days after the first dose. Serious adverse events were recorded for the duration of the study. Clinical chemistry tests (alanine aminotransferase and creatinine) and haematology tests (complete blood count, with differential for neutrophils and lymphocytes only) were done at screening and 7 days after the first immunisation.

Toxicity grading of clinical and laboratory variables and vomiting was done according to the Division of AIDS Table for Grading the Severity of Adult and Paediatric Adverse Events, 2009, of the US National Institutes of Health.^19^ Dehydration was assessed and managed as per the WHO Integrated Management of Childhood Illness guideline.^20^ Diarrhoea was defined as at least three unformed or loose stools in a 24-h period or at least one bloody loose or liquid stool or one to two liquid stools with at least some signs of dehydration in a 24-h period and during the home visits 5–7 days after each vaccination. Loose stool was defined as fewer than three episodes of unformed stools with mixed liquid and solid components in a 24-h period and during home visits.

For measurement of immune responses, heparinised venous blood samples (2–4 mL) were obtained 4–7 days before the first immunisation, 7 days after the first dose, and 5 days after the second dose (day 19). We have identified these timepoints to be optimal for assessing circulating antibody-secreting cell or ALS responses after oral vaccination, with only minor differences in plasma IgA and IgG responses between day 5 and later timepoints after the second dose.^16^, ^21^, ^22^ Immune responses were assessed by measuring vaccine-specific (ie, CFA/I, CS3, CS5, CS6, and LTB) IgA antibodies in ALS specimens^16^, ^18^, ^23^ and IgA antibody levels against the five vaccine antigens and IgG antibody concentrations against LTB in plasma.^16^, ^23^, ^24^ Peripheral blood mononuclear cells (PBMCs) and plasma were separated from the blood by density-gradient centrifugation on Ficoll-Isopaque. Plasma specimens were preserved at −20°C. For ALS preparations, 10^7^ PBMCs per mL were cultured for 48 h at 37°C with 5% CO_2_; supernatants were collected and stored at −70°C.^16^, ^18^, ^22^

To enable analysis of ALS responses to all primary vaccine antigens in the small sample volumes available from children, a highly sensitive electrochemiluminescence assay was established based on Meso Scale Discovery (Rockville, MD, USA) technology. Electrochemiluminiscence and ELISA results were shown to be highly correlated in adults.^18^ For the ELISA and electrochemiluminiscence assays, CFA/I was prepared from a rough *E coli* strain^25^ at University of Gothenburg, Sweden, and CS3 from an O139 lipopolysaccharide *E coli* strain and CS5 from an O167 lipopolysaccharide *E coli* strain at Scandinavian BioPharma; the CS6 antigen was provided by F Cassels (National Institutes of Health, USA). LTB was produced at Scandinavian BioPharma. The CFA/I antigen was O antigen free, and the CS5, CS6, and LTB antigens contained only trace amounts of lipopolysaccharide as analysed by silver staining. Because silver staining indicated low levels of O139 lipopolysaccharide in the CS3 antigen, a CS3 antigen containing only trace amounts of lipopolysaccharide (100 pg lipopolysaccharide per mg protein) provided by the National Institutes of Health was used for control experiments.

Faecal samples were collected 4–7 days before the first immunisation and then 7, 19, and 28 days after the first immunisation; specimens were extracted as described previously^23^, ^26^ and stored at −70°C. Total secretory IgA concentrations were measured by ELISA after the first and second doses on days 7, 19, or 28 (or a combination of these days) using a secretory IgA reference (Sigma-Aldrich, St Louis, MO, USA).^23^, ^26^ Samples containing less than 10 μg/mL or more than 1000 μg/mL total secretory IgA and samples in which the total secretory IgA concentration varied by more than a three-times difference between the different timepoints for each participant were excluded from the analysis.^16^, ^27^ Faecal extracts were assayed for secretory IgA antibody responses against colonisation factors and LTB by ELISA; secretory IgA antibody titres were divided by the total secretory IgA concentration of each sample.^16^, ^26^ Most (60–90%) of the stool samples from children aged 12 months or older contained low and variable concentrations of total secretory IgA. Therefore, only stool samples from children aged 6–11 months were assessed for secretory IgA antibody responses. Low and variable concentrations of secretory IgA in faecal samples were also recorded in adults^18^ and in a previous study of cholera vaccine in older children in the same area.^21^ Stool specimens from infants were analysed for lactoferrin concentrations (Innovative Research Kit, Peary Court Novi, MI, USA) to exclude that responses were caused by breast milk contamination (appendix p 14).

### Outcomes

The primary outcome was safety. Primary safety endpoints were number of serious adverse events, adverse events, and vaccine-induced reactogenicity events. Secondary immunology endpoints were antibody responses (at least two-times increase in antibody levels between baseline and post-immunisation) and geometric mean titres and geometric mean fold rises between baseline and post-immunisation in antibodies to CFA/I, CS3, CS5, CS6 and LTB, as measured in lymphocyte secretions (for IgA), faecal samples (for secretory IgA, in infants only), and plasma samples (for IgA and IgG). Additionally, as a secondary endpoint, we measured antibody responses and geometric mean titres and geometric mean fold rises in antibodies to O78 lipopolysaccharide; the results of these analyses will be reported elsewhere. Exploratory endpoints were T-cell immune responses, neutralising antibody geometric mean titres, avidity of immune responses, immunoproteomic profile of vaccine-induced antibody responses in plasma or intestinally derived samples, and crossreactive immune responses against vaccine-related colonisation factors, and will be reported elsewhere.

### Statistical analysis

The sample size for this study was selected to detect frequent adverse events. Given a planned sample size of groups of 15 adults, toddlers, and younger children, the study would have about an 80% chance of observing at least one serious adverse event for events that occur at a rate of 10·3% and a 90% chance of observing one adverse event of special interest at a rate of 14·3%. Additionally, if no serious adverse events are observed in 15 participants, the upper bound of the one-sided 95% CI on the rate of serious adverse event occurrence would be about 18%. For infant cohorts with 30 participants per dose group, the study would have about an 80% chance of observing at least one serious adverse event for events that occur at a rate of 5·3% and a 90% chance of observing one adverse event of special interest at a rate of 7.4%. If no serious adverse events are observed in 30 participants, the upper bound of the one-sided 95% CI on the rate of serious adverse event occurrence would be about 9·5%.

The magnitudes of immune responses (fold rises) were calculated as the post-immunisation response (the highest response after the first or second dose) divided by pre-immunisation antibody levels, and at least two-times increases were regarded as responses.^16^, ^28^ Immune responses against vaccine antigens were compared between vaccine and placebo recipients with unpaired Student's *t* test, using log-transformed data with Holm's Bonferroni adjustments for analyses of responses against multiple antigens. Proportions of participants with at least two-times increases in antibody levels between baseline and post-immunisation (ie, responder frequencies) were compared between groups using Fisher's exact test. The Holm-Bonferroni method established the family-wise error rate for all statistical comparisons against each antigen stratified by visit day and age group. A multivariable logistic regression model was used to assess the odds of vomiting in the first 24 h after the first or second dose by fitting age, vaccine dose, and dmLT dose to the model. Adjusted odds ratios were obtained by simultaneously adding the three variables (age, vaccine dose, and dmLT dose) to the model as categorical variables or, in the case of trend test, uncategorised. Adjusted odds ratios, 95% CIs, and p values were obtained from model parameters. Model calibration was assessed by the Hosmer-Lemeshow goodness-of-fit test, with p-values greater than 0·05 indicating an adequate fit. All statistical analyses were done with GraphPad Prism, version 7.0, and SAS, version 9.4. p values had to be less than 0·05 to be considered significant. Safety data were reviewed by an independent protocol safety team before dose escalation and age de-escalation, and by the International Centre for Diarrhoeal Disease Research, Bangladesh, data safety monitoring board before age de-escalation. This trial is registered with ClinicalTrials.gov (NCT02531802).

### Role of the funding source

The funder participated in the design of the study, data interpretation, and writing of the report, but not data collection or data analysis. The corresponding author had full access to all the data in the study and final responsibility for the decision to submit for publication.

## Results

Between Dec 7, 2015, and Jan 10, 2017, we screened 1500 children across the three age groups, of whom 430 were enrolled and randomly assigned to the different treatment groups (130 children aged 24–59 months, 100 aged 12–23 months, and 200 aged 6–11 months; table 1; appendix pp 2–4). The sex, age, and nutritional status distributions of children were similar between the different cohorts in each age group (appendix p 5). All children were from a similar socioeconomic background.

**Table 1:** Group assignment

		**N (planned)**	N (actual)*
24–59 months		150	130
	Quarter-dose ETVAX	15	15
	Half-dose ETVAX	15	15
	Full-dose ETVAX	15	3
	Half-dose ETVAX plus 2·5 μg dmLT	15	15
	Half-dose ETVAX plus 5·0 μg dmLT	15	15
	Half-dose ETVAX plus 10·0 μg dmLT	15	15
	Placebo	60	52
12–23 months		100	100
	Quarter-dose ETVAX	15	15
	Half-dose ETVAX	15	15
	Half-dose ETVAX plus 2·5 μg dmLT	15	15
	Half-dose ETVAX plus 5·0 μg dmLT	15	15
	Placebo	40	40
6–11 months		200	200
	Eighth-dose ETVAX	30	30
	Quarter-dose ETVAX	30	30
	Half-dose ETVAX	30	30
	Quarter-dose ETVAX plus 2·5 μg dmLT	30	30
	Quarter-dose ETVAX plus 5·0 μg dmLT	30	30
	Placebo	50	50

ETVAX=enterotoxigenic *Escherichia coli* vaccine. dmLT=double-mutant heat-labile toxin.

* Safety analysis population.

Across all age groups, no solicited adverse events occurred that were greater than moderate in severity, and most were mild (table 2). The most common solicited adverse event was vomiting. Most episodes were mild, with moderate vomiting only in participants given the largest fractional doses. Vomiting was inversely related to age (p=0·004) and correlated to vaccine dose, with lower doses presenting less risk of vomiting (p=0·0076; appendix p 6). In children aged 24–59 months, moderate vomiting was observed in two of the three children receiving the full dose of vaccine. Because the study was not designed to test the full dose in children, although one of the goals of the study was to establish the highest tolerable dose, enrolment in this cohort was curtailed on the basis of vomiting events, identifying the half dose as the largest tolerated dose for children aged 24–59 months, as well as for children aged 12–23 months. Ten of 30 infants aged 6–11 months given the half dose had vomiting, thus the quarter dose was the largest tolerated dose to test in combination with dmLT in this age group. The addition of dmLT did not appear to modify the frequency or severity of vomiting (p=0·10; appendix p 6). Vomiting occurred exclusively in vaccine recipients, generally started within 1–8 h after administration, and was transient (in all cases it resolved without intervention within 1 day).

**Table 2:** Solicited adverse events

		**Fever**	**Vomiting**	**Loose stools**	**Diarrhoea**
**24–59 months**					
All (n=130)		6 (5%)	10 (8%)	0	1 (1%)
	Quarter-dose ETVAX (n=15)	0	2 (13%)	0	0
	Half-dose ETVAX (n=15)	1 (7%)	0	0	0
	Full-dose ETVAX (n=3)	0	2 (67%)*	0	0
	Half-dose ETVAX plus 2·5 μg dmLT (n=15)	3 (20%)†	2 (13%)	0	0
	Half-dose ETVAX plus 5·0 μg dmLT (n=15)	0	1 (7%)	0	0
	Half-dose ETVAX plus 10 μg dmLT (n=15)	0	3 (20%)	0	0
	Placebo (n=52)	2 (4%)	0	0	1 (2%)
**12–23 months**					
	All (n=100)	6 (6%)	13 (13%)	3 (3%)	5 (5%)
	Quarter-dose ETVAX (n=15)	1 (7%)†	2 (13%)	0	1 (7%)
	Half-dose ETVAX (n=15)	0	1 (7%)	0	1 (7%)
	Half-dose ETVAX plus 2·5 μg dmLT (n=15)	3 (20%)†	2 (13%)	3 (20%)	0
	Half-dose ETVAX plus 5·0 μg dmLT (n=15)	1 (7%)	8 (53%)	0	2 (13%)
	Placebo (n=40)	1 (3%)	0	0	1 (3%)
**6–11 months**					
All (n=200)		17 (9%)	29 (15%)	4 (2%)	8 (4%)
	Eighth-dose ETVAX (n=30)	2 (7%)	2 (7%)	0	0
	Quarter-dose ETVAX (n=30)	2 (7%)	5 (17%)	1 (3%)	1 (3%)
	Half-dose ETVAX (n=30)	2 (7%)†	10 (33%)*	1 (3%)	2 (7%)
	Quarter-dose ETVAX plus 2·5 μg dmLT (n=30)	2 (7%)	7 (23%)	0	1 (3%)
	Quarter-dose ETVAX plus 5 μg dmLT (n=30)	3 (10%)	5 (17%)	0	1 (3%)
	Placebo (n=50)	6 (12%)*	0	2 (4%)	3 (6%)

All events were mild unless otherwise indicated. No participants had abdominal pain or stomachache or acute systemic allergic reaction. Moderate vomiting was defined as more than five episodes within a 24-h period. Moderate fever was defined as non-axillary temperature 38·7–39·3°C. ETVAX=enterotoxigenic *Escherichia coli* vaccine.

* Two participants had moderate adverse events.

† One participant had a moderate adverse event.

In all age groups, the only other solicited event that was ever greater than mild in severity was fever (table 2). Moderate fever occurred in at most one participant in any vaccine dose group, and the frequency of fever did not differ significantly between vaccine and placebo groups. 29 (7%; 20 children who received the vaccine and nine who received placebo) of the 430 participants had solicited fever, of which the majority occurred at least 24 h after vaccine administration and for a short duration. Only two participants (one in the age 6–11 months quarter dose and one in the age 6–11 months quarter dose with 2·5 μg dmLT group) had fever on the day of vaccination, and in both the fever was mild and resolved on the same day. The other solicited events were infrequent and mild (table 2).

As part of routine safety monitoring, blood tests were done on all participants 7 days after vaccination. The laboratory aberrations detected during monitoring that were assessed by investigators as treatment-related included seven events, most of which were increased white blood cell count. The investigators were unaware of treatment assignment when assessing relatedness to treatment. Of note, four of those seven events were in placebo recipients; given the ratio of vaccine to placebo recipients, the proportion of events in vaccine recipients was lower than in placebo recipients. Non-serious, unsolicited adverse events recorded during the short period of observation were infrequent and without discernible pattern in terms of nature of event or product received (appendix pp 7–9). The data from safety monitoring tests were unremarkable, without discernible patterns or differences between children receiving vaccine and those receiving placebo (appendix pp 7–9).

Three serious adverse events were observed in three children: one child aged 24–59 months who received placebo had fever followed by diarrhoea 13 days after the first dose and was admitted to hospital with enteric fever, one child aged 12–23 months who received a quarter dose of ETVAX was admitted to hospital for rickets on day 46 after first vaccination, and one infant aged 6–11 months who received a half dose of ETVAX was admitted to hospital for pneumonia on study day 15. No serious adverse event was considered related to the study drug.

**Figure 1:**
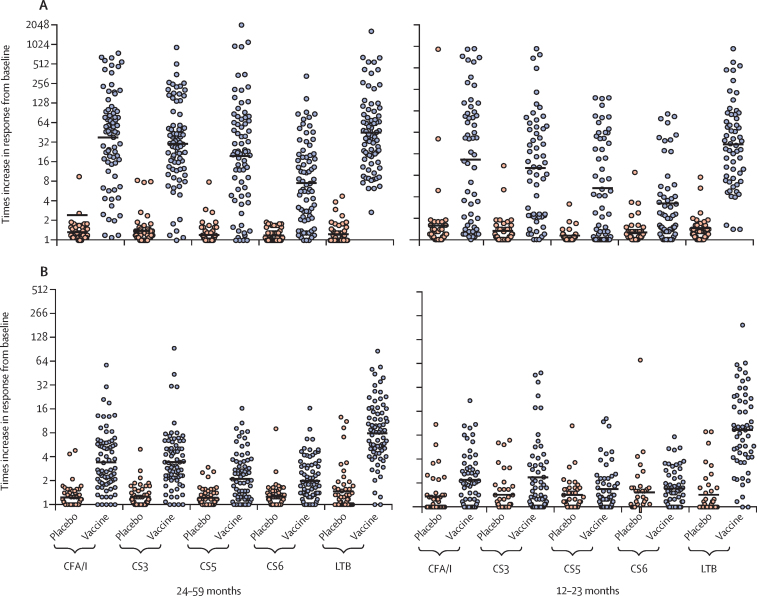
Geometric mean times increases in ALS (A) and plasma (B) IgA responses in children aged 12–59 months Horizontal lines indicate the geometric mean response for the group, whereas the circles represent individual responses. Times increases were calculated as the post-immunisation response (the highest response on either day 7 after the first dose or day 5 after the second dose) divided by pre-immunisation antibody level. Geometric mean times increases in ALS concentrations were significantly different between vaccine and placebo recipients for all antigens in both age groups (all p<0·0001 with Student's *t* test and all p<0·0005 with Holm's-Bonferroni adjustment). In children aged 24–59 months, geometric mean times increases in plasma IgA concentrations were significantly different between vaccine (n=75) and placebo (n=50) recipients for all antigens (all p<0·0001 with Student's *t* test and all p=0·0005 with Holm's-Bonferroni adjustment); in children aged 12–23 months, the difference between vaccine (n=60) and placebo (n=40) recipients was significant only for CFA/I, CS3, and LTB (all p≤0·0061 with Student's *t* test and all p≤0·018 with Holm's-Bonferroni adjustment). ALS=antibodies in lymphocyte secretions.

Analysis of ALS specimens in children aged 24–59 months (excluding the three children who received the full vaccine dose) showed that two immunisations with ETVAX alone (quarter or half dose) or half doses with different doses of dmLT elicited high and significant (ie, at least two-times increase versus baseline) IgA responses against all four vaccine colonisation factors and LTB, as measured 7 days after the first dose or 5 days after the second dose; few responses were observed in placebo recipients (appendix p 10). Addition of dmLT to the vaccine did not have a significant effect on the ALS responses, and no significant differences in responses against any of the five vaccine antigens were noted among the different cohorts (appendix p 10). The magnitudes of responses in this age group (when combining responses in the five cohorts) were all highly significant and similar for CFA/I, CS3, CS5, and LTB (18–50-times increase), with somewhat lower responses against CS6 (eight-times increase; figure 1A). Responder frequencies for vaccine recipients in this age group ranged from 61 (81%) of 75 for CS6 to 75 (100%) for LTB (appendix p 11).

Analysis of ALS specimens in the different cohorts of children aged 12–23 months showed that two immunisations with ETVAX alone (quarter or half dose) or half doses with 2·5 μg or 5·0 μg dmLT elicited significant increases in IgA antibodies in most vaccinees against all four vaccine colonisation factors and LTB (appendix p 12). These responses were of somewhat lower magnitudes than those observed in the older children. The magnitudes of responses in the different cohorts were similar against CFA/I, CS3, and LTB (six to 20-times increases), but somewhat lower against CS5 and CS6 (three to six times increases; appendix p 12). Addition of dmLT to the vaccine did not have any significant effect on the ALS responses (appendix p 12). Combined responses in all four cohorts showed that most vaccinees (ranging from 29 [51%] of 57 children for CS6 to 51 [95%] for LTB), but only a few placebo recipients (ranging from one [3%] of 40 children for CS5 to six [16%] for CS6), had at least two times increases in concentrations of antibodies to the vaccine antigens, and the magnitudes of responses and responder frequencies were significantly higher in vaccinees than in placebo recipients against each antigen (figure 1A; appendix p 11).

As in previous studies,^10^ ALS responses against the vaccine antigens in infants aged 6–11 months were low but significantly higher in vaccinees than placebo recipients for CFA/I (54 [38%] of 144 infants who received vaccine *vs* eight [16%] of 50 who received placebo; p=0·015), CS3 (58 [40%] *vs* four [8%]; p<0·0005), and LTB (107 [74%] *vs* 14 [28%]; p<0·0005; appendix p 13). Because immune responses did not significantly differ by vaccine dose among infants aged 6–11 months, an analysis of faecal immune responses as a more direct approach to assess intestinal immune responses was also done for all infants in this age group (pooled data from all cohorts) who provided faecal extracts that fulfilled inclusion criteria (ie, with concentrations of secretory IgA that were within detection limits and did not differ more than three-fold between different timepoints; n=177).

**Figure 2:**
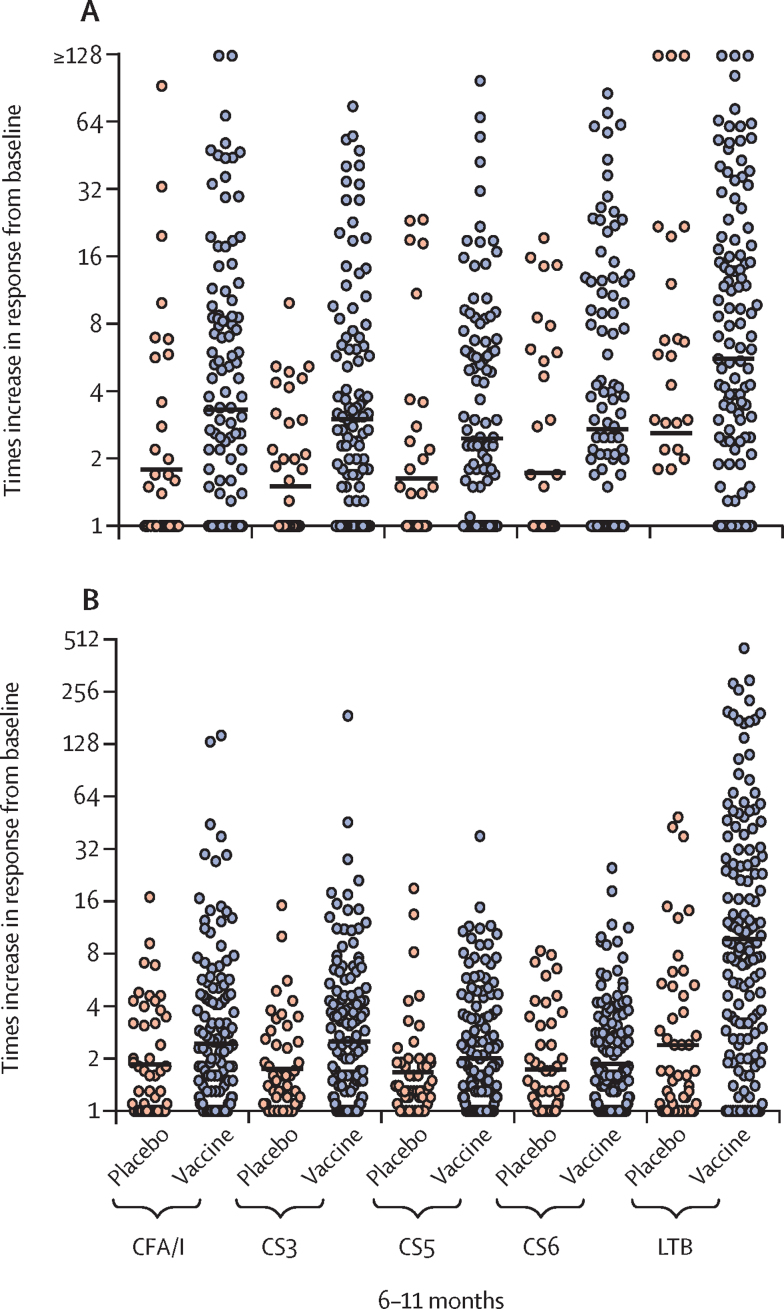
Geometric mean times increases in faecal secretory IgA (A) and plasma IgA (B) responses in infants aged 6–11 months Horizontal lines indicate the geometric mean response for the group, whereas the circles represent individual responses. Times increases were calculated as the post-immunisation response (the highest response on either day 7 after the first dose or day 5 or 14 after the second dose) divided by pre-immunisation antibody levels. Faecal secretory IgA responses were the ratio between antigen-specific faecal secretory IgA antibody titres divided by total secretory IgA in preimmunisation and corresponding postimmunisation samples. Significant differences between vaccine (n=131) and placebo (n=46) recipients in faecal secretory IgA times increases were observed for CFA/I (p=0·0050), CS3 (p=0·0005), CS5 (p=0·026), CS6 (p=0·028), and LTB (p=0·0020) before Holm's-Bonferroni adjustment and for CFA/I (p=0·015), CS3 (p=0·0025), and LTB (p=0·0080) after adjustment. Significant differences in geometric mean times increases in plasma IgA between vaccine (n=148) and placebo (n=50) recipients were seen for CS3 (p=0·013) and LTB (p<0·0001) before Holm's-Bonferroni adjustment and for LTB (p<0·0001) after adjustment.

The magnitudes of faecal secretory IgA responses against all antigens in infants aged 6–11 months were significantly higher in vaccinees than placebo recipients (only against CFA/I, CS3, and LTB after Holm's Bonferroni adjustment), even in infants without a positive ALS response (figure 2A). Lactoferrin concentration was analysed in faecal specimens to exclude the possibility that responses were related to breast milk contamination. Few lactoferrin responses were observed in infants who received vaccine and responded to CFA/I and LTB (appendix p 14); results were similar for those responding to the other colonisation factor antigens and across the other infant cohorts (data not shown). The few infants with faecal IgA antibody responses to colonisation factors or LTB who also showed increases in their faecal lactoferrin concentrations had considerably lower increases in lactoferrin concentrations than in antibody responses (appendix p 14), suggesting that the noted antigen-specific increases resulted from the immunisation and were not related to the ingestion of breast milk antibodies. A secondary endpoint in the protocol was responses by either mucosal measure; this analysis showed that most of the vaccinated infants had significant mucosal immune responses (ALS IgA or faecal secretory IgA, or both) to the colonisation factors (from 70 [49%] of 144 for CS5 and CS6 to 89 [62%] for CS3) and LTB (116 [81%]; appendix p 15).

Colonisation factor-specific IgA and LTB-specific IgA and IgG responses in plasma were measured before immunisation and 7 days after the first and 5 days after the second dose (post-immunisation results given are the highest response on either of these days). In children aged 24–59 months, these analyses showed that two immunisations with quarter or half doses of ETVAX or half doses with different doses of dmLT elicited significant IgA responses against most vaccine antigens in most children (appendix p 16). IgG responses to LTB were also induced but of lower magnitude than LTB IgA responses. No apparent differences were observed between responses to the different doses of vaccine or to vaccine given with and without dmLT (appendix p 16). Few placebo recipients responded, and the frequencies and magnitudes (lowest for CS5 and CS6) of plasma responses were lower in vaccinees than observed for ALS responses (figure 1B; appendix pp 16, 19).

Colonisation factor-specific and LTB-specific IgA antibody responses in plasma from the children aged 12–23 months showed that two immunisations with quarter or half doses of ETVAX without dmLT or half doses with 2·5 μg or 5·0 μg dmLT elicited significant (at least two-times) increases in IgA antibody responses to most vaccine antigens, except CS5 and CS6, in most children (appendix p 17). Similar to the older children, IgG responses to LTB were induced in most vaccinees but with smaller increases than corresponding IgA responses. Plasma responses among vaccine recipients in this age group were significantly higher for CFA/I, CS3, and LTB, and significantly more frequent for CFA/I and LTB, than in placebo recipients (figure 1B; appendix p 19).

Colonisation factor-specific IgA and LTB-specific IgA and IgG responses in plasma specimens from infants aged 6–11 months showed that eighth, quarter, and half doses of ETVAX induced at least two-times increases in IgA and IgG immune responses against LTB when comparing antibody levels before and after vaccination. The frequency and magnitude of anti-LTB responses to all doses were similar. Addition of dmLT to the quarter dose did not have an apparent effect on immune responses to any antigen (appendix p 18). Many of the placebo recipients also had responses to the vaccine antigens, possibly because of asymptomatic enterotoxigenic *E coli* infections during the study period. Despite this finding, when combining results for all dose cohorts, antibody responses to all vaccine antigens tended to be higher in the vaccine group than the placebo group but were only significantly increased and more frequent for LTB (p=0·0005; figure 2B; appendix p 19).

**Figure 3:**
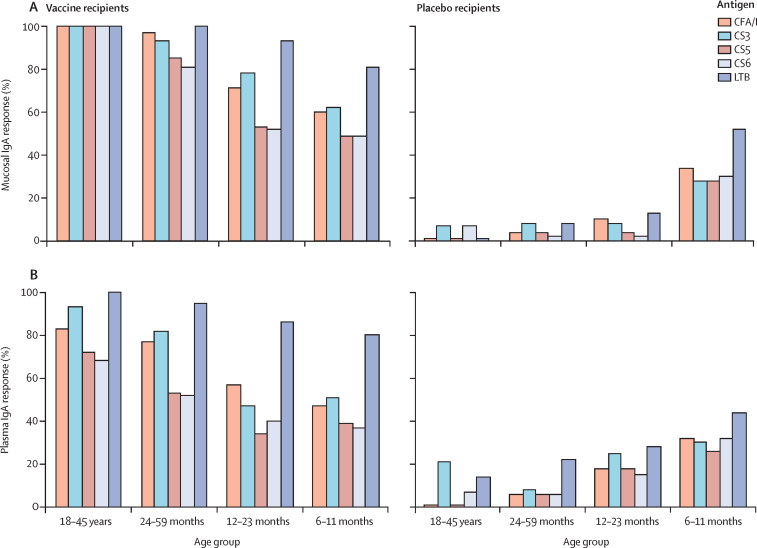
Frequencies of mucosal IgA (ALS or faecal, or both) and plasma IgA responses in all age groups Frequencies of mucosal (A) and plasma (B) IgA antibody responses against the five primary vaccine antigens in different age groups in Bangladesh. Adult data from the same protocol included for comparison.^18^ All participants received two doses of ETVAX with or without dmLT or two doses of placebo. Mucosal responses were measured in ALS specimens for adults and children aged 12–59 months and in ALS or faecal specimens for infants aged 6–11 months. ALS=antibodies in lymphocyte secretions. ETVAX=enterotoxigenic *Escherichia coli* vaccine.

Immune responses to the vaccine, with or without dmLT, were age related. The frequencies of ALS IgA responses against each colonisation factor declined with decreasing age of the vaccinees, whereas responses against LTB were almost similar across the age groups (figure 3; appendix pp 11, 13). Conversely, mucosal responses in the placebo recipients declined with increasing age (figure 3; appendix pp 11, 13, 19). Plasma responses followed a similar age-related pattern (figure 3; appendix p 19). The breadth of immune responses (ie, frequency of responders to different numbers of antigens) was greater in ALS (for children aged 12–59 months) and faecal samples (for infants aged 6–11 months) than in plasma samples and increased with age (table 3).

**Table 3:** IgA antibody responses to different numbers of ETVAX antigens

	**24–59 months**		**12–23 months**		**6–11 months**	
	Vaccine (n=75)	Placebo (n=49)	Vaccine (n=54)	Placebo (n=40)	Vaccine (n=139)	Placebo (n=49)
**Mucosal IgA**						
Five antigens	56 (75%)	0	16 (30%)	0	32 (23%); 6 (4%)	4 (8%); 0
Four antigens	68 (91%)	0	30 (56%)	2 (5%)	51 (37%); 19 (14%)	12 (24%); 1 (2%)
Three antigens	72 (96%)	1 (2%)	41 (76%)	2 (5%)	78 (56%); 46 (33%)	14 (29%); 1 (2%)
Two antigens	75 (100%)	3 (6%)	48 (89%)	4 (10%)	107 (77%); 78 (56%)	22 (45%); 8 (16%)
One antigen	75 (100%)	9 (18%)	52 (96%)	9 (23%)	133 (96%); 117 (84%)	41 (84%); 22 (45%)
None	0	40 (82%)	2 (4%)	31 (78%)	6 (4%); 22 (16%)	8 (16%); 27 (55%)
**Plasma**						
Five antigens	21 (28%)	2 (4%)	10 (19%)	2 (5%)	33 (24%)	4 (8%)
Four antigens	42 (56%)	2 (4%)	16 (30%)	4 (10%)	47 (34%)	10 (20%)
Three antigens	65 (87%)	2 (4%)	26 (48%)	6 (15%)	65 (47%)	14 (29%)
Two antigens	70 (93%)	5 (10%)	38 (70%)	8 (20%)	89 (64%)	22 (45%)
One antigen	74 (99%)	14 (29%)	52 (96%)	19 (48%)	122 (88%)	31 (63%)
None	1 (1%)	35 (71%)	1 (2%)	21 (53%)	17 (12%)	18 (37%)

Data are n (%). ALS responses on day 7 or day 19 are shown for children aged 12–59 months; for infants aged 6–11 months, mucosal IgA data are presented as faecal secretory IgA responses followed by ALS responses (below). A response was defined as at least a two-times increase in antibody levels between preimmunisation and days 7 or 19. ALS=antibodies in lymphocyte secretions. ETVAX=enterotoxigenic *Escherichia coli* vaccine.

Comparison of ALS immune responses to the first and second vaccine doses showed that most of the children in the different age groups responded to the first dose. The only significantly higher responses to the second dose versus the first dose were against CS6 in children aged 24–59 months (p=0·015) and against CS3 (p=0·0041) and LTB (p<0·0001) in infants (appendix p 20).

Analysis of faecal immune responses in infants aged 6–11 months who were given a quarter dose of vaccine plus 2·5 μg of dmLT showed that dmLT significantly improved the faecal secretory IgA responses to three of the five antigens after the first immunisation (ie, day 7; appendix p 21). Two doses of vaccine without dmLT were needed to induce responses that were similar to those seen among infants receiving one dose of vaccine with dmLT. Furthermore, there was an increased frequency of mucosal antibody responses (ALS or faecal, or both) to three or more vaccine antigens in infants who received the vaccine with dmLT compared with infants receiving the same dose of vaccine alone (appendix p 22). Although the breadth of the response appeared greater in infants who received the adjuvanted vaccine than in those who received the vaccine without adjuvant, these differences were not significant. When comparing the breadth of response in the two vaccine groups with that in the placebo group, significantly more infants in the vaccine plus dmLT group than in the placebo group had antibody responses to three, four, or five antigens. In the vaccine-only group, antibody responses to multiple antigens were significant compared with placebo only for number of children responding to all five antigens (appendix p 22), although this response was of low frequency compared with infants given the vaccine with dmLT (seven [23%] of 30 without dmLT *vs* 12 [43%] of 28 with dmLT). When a more stringent measure (ie, at least a four-times increase in mucosal antibody concentration) was used, dmLT strengthened the response to all colonisation factor antigens in infants, but only the CFA/I response was significant (appendix p 23).

## Discussion

To the best of our knowledge, this is the first clinical trial of ETVAX, as well as of the dmLT adjuvant, in children and infants. The primary objective was to ascertain safety data for ETVAX with or without dmLT and to identify the largest tolerable dose of vaccine and adjuvant in different age groups of Bangladeshi children. We show that two fractionated doses of ETVAX, when administered with or without different doses of dmLT, were safe and generally well tolerated in children. The most prevalent adverse events recorded were mild and, in a few cases, moderate vomiting, which is similar to what was observed in a previous study in young children in Bangladesh receiving similar amounts of inactivated enterotoxigenic or non-enterotoxigenic *E coli* bacteria.^24^ At a quarter (2·5 × 10^10^ cells) of an adult dose, 13–17% of children in any of the three age cohorts had vomiting, which was mild in all cases. In infants aged 6–11 months, the dose was further reduced to an eighth of an adult dose, which resulted in a reduced frequency of vomiting (7% of infants). The doses of dmLT tested (2·5–10·0 μg) did not affect the safety or tolerability of the vaccine, as shown by multivariate analysis (appendix p 6).

Fractional doses of the vaccine reduced vomiting while remaining immunogenic. For example, in the youngest age group, immune responses at an eighth of an adult dose were similar to those in children given higher doses. These findings, together with the reduced vomiting at lower doses, indicate that infants might be safely immunised with low doses of ETVAX without unacceptable loss of immunogenicity.

High and frequent ALS immune responses to all five primary vaccine antigens were observed using the electrochemiluminiscence method in all vaccine groups except in the youngest age group. For the older age groups, the high magnitudes of ALS responses in some of the placebo recipients against CFA/I, CS3, CS6, and LTB might suggest asymptomatic infection with enterotoxigenic *E coli* or infection with vibrios expressing cholera toxin during the study period. ALS responses against the colonisation factors among vaccinated infants were infrequent (15–40%), which is in agreement with studies on oral cholera vaccine^21^ and rotavirus vaccine in Indian children.^29^ The study on rotavirus vaccine^29^ showed that only 68 (26%) of 258 of children who did not have increased rotavirus-specific IgA antibody-secreting cell levels at onset of vaccination developed significant IgA antibody-secreting cell responses to the rotavirus vaccine despite the same vaccine inducing protection in 45% of infants in similar populations in Bangladesh.^30^ These results suggest that measuring circulating antibody-secreting cell or ALS responses is not optimal for assessment of mucosal immune responses to oral enteric vaccines in very young children. In this study, we show that secretory IgA anti-colonisation factor antibody responses in faecal extracts were considerably more frequent in infants than were ALS IgA responses to the same antigens. We identified frequent faecal immune responses in infant placebo recipients, which might suggest high incidence of asymptomatic infections in young children in high-endemic areas.^2^

The first generation of an oral inactivated ETEC vaccine consisted of a mixture of five different formalin-inactivated strains expressing CFA/I and CS1–5, but including considerably lower amounts of CFA/I, CS3, and CS5 than in ETVAX; CS6 was not present and the toxoid component was CTB and not LCTBA. It was tested in Swedish, US, and Egyptian adults, as well as Bangladeshi and Egyptian children and infants.^10^ Based on WHO recommendations, the initial formulation was improved upon with this version of ETVAX and the first generation was not assessed beyond the phase 2/3 trials in Bangladesh and Egypt and the phase 3 trials in Mexico and Guatemala (US travellers). An in-depth comparison of plasma and mucosal responses across studies of the first generation and second generation of ETVAX is not meaningful because of the different methods used, the different timepoints at which responses were assessed after the second dose, and the different spectrum and purity of vaccine antigens. However, a superficial comparison of the these vaccines in Bangladeshi infants and children suggests they induced similar frequencies and magnitudes of plasma IgA responses to the toxoid components.^24^, ^31^

Our data suggest that any immunological effect of reduced doses of vaccine in infants might be mitigated by coadministration of the dmLT adjuvant. Furthermore, dmLT improved the kinetics of faecal secretory IgA responses—ie, responses to the adjuvanted vaccine were induced after the first dose, whereas a similar response to the non-adjuvanted vaccine was induced after only two doses. When a definition of an at least four-times increase for responses was applied, the apparent effect of dmLT coadministration with the vaccine was greater (appendix p 23).

Immune responses to ETVAX in infants might be further improved by administration of three rather than two doses. Beneficial effect of a third dose has been seen in a previous field trial of rotavirus vaccine in South Africa.^32^ In our study, we noted that responses to CS3 and LTB were significantly higher after the second dose than after the first dose in infants, suggesting that the effect of a third dose should be assessed in this age group in future studies. Encouragingly, responses to most antigens (other than CS3 and LTB) were remarkably robust, especially with the dmLT adjuvant, even after a single vaccine dose.

An important finding is that Bangladeshi infants receiving ETVAX mounted significant mucosal immune responses to all primary ETVAX antigens. Children in LMICs have been difficult to effectively immunise with other oral vaccines—eg, cholera and rotavirus vaccines.^21^, ^29^, ^33^ Based on the positive effect of dmLT in infants, further analysis of the adjuvanted vaccine is justified. Phase 1 and 2b trials based on the doses established in the youngest children in this study, an eighth or quarter dose of vaccine plus 2·5 μg dmLT, has been initiated in children in Zambia and will be followed by studies in The Gambia. Additional studies based on the work described here will not only be important for confirmation of ETVAX's public health benefit but could also enable more effective use of other oral inactivated vaccines in infants.

A strength of this study was the use of the electrochemiluminescence assay to assess mucosal immune responses against vaccine antigens in the small sample volumes that were available from children. The mucosal immune response data were strengthened by use of optimal timepoints^21^, ^22^ for assessing ALS responses and by analysing faecal secretory IgA immune responses in infants. We have few missing data, but a limitation of this study is our inability to analyse faecal immune responses in older children because faecal samples from children older than 1 year did not fulfil predefined criteria for analysis. Also, we were not able to test dmLT with the eighth dose. Furthermore, because this is the first study done in a cohort of Bangladeshi children, it is unknown whether the results can be generalised to other populations.

In conclusion, this study established the largest tolerated dose of ETVAX in different age groups of Bangladeshi children and the possible benefits of dmLT. This was a single-centre study in Bangladesh, and we have now initiated a phase 1 study of ETVAX in Zambia that will be followed by a phase 2b study in The Gambia to assess safety, immunogenicity, and protective efficacy of ETVAX in young children in different settings.

## Data sharing

The data are not publicly available because of ethical restrictions on participant privacy, but data from this study will be made available to others in the scientific community upon request after the ongoing phase 2b study of ETVAX in Finnish travellers (NCT03729219) has been completed and the scientific data from the development of this candidate have been fully published. Standard criteria for making data available for valid research projects will be used, following application by suitably qualified researchers. All publications coming from the clinical and field evaluations of the ETVAX vaccine will appear in journals that have an open access format.
