# Supplementary material for: Safety and immunogenicity of the oral, inactivated, enterotoxigenic Escherichia coli vaccine ETVAX in Bangladeshi children and infants: a double-blind, randomised, placebo-controlled phase 1/2 trial
Source: Lancet Infect Dis. 2020 Feb;20(2):208–19. doi: 10.1016/S1473-3099(19)30571-7 (PMC6990395; doi:10.1016/S1473-3099(19)30571-7)
Supplement: Supplementary appendix [file mmc1.pdf]

THE LANCET Infectious Diseases

Supplementary webappendix

This webappendix formed part of the original submission and has been peer reviewed. We post it as supplied by the authors.

Supplement to: Qadri F, Akhtar M, Bhuiyan TR, et al. Safety and immunogenicity of the oral, inactivated, enterotoxigenic *Escherichia coli* vaccine ETVAX in Bangladeshi children and infants: a double-blind, randomised, placebo-controlled phase 1/2 trial. *Lancet Infect Dis* 2019; published online Nov 19. https://doi.org/10.1016/S1473-3099(19)30571-7.

Appendix 1. Study groups in ETVAX vaccine trial in Bangladesh (OEV-122/VAC 014)

	Cohort	ETVAX	dmLT	N*
**PART B: 24-59 months, inclusive**	B1	¼ dose	-----	15
		Placebo	-----	10
	B2	½ dose	-----	15
		Placebo	-----	10
	B3	Full dose	-----	15
		Placebo	-----	10
	B4	½ dose	2.5 µg	15
		Placebo	-----	10
	B5	½ dose	5 µg	15
		Placebo	-----	10
	B6	½ dose	10 µg	15
		Placebo	-----	10
			Total	150

	Cohort	ETVAX	dmLT	N*
**PART C: 12-23 months, inclusive**	C1	¼ dose	-----	15
		Placebo	-----	10
	C2	½ dose	-----	15
		Placebo	-----	10
	C3	½ dose	2.5 µg	15
		Placebo	-----	10
	C4	½ dose	5 µg	15
		Placebo	-----	10
			Total	100

	Cohort	ETVAX	dmLT	N*
**PART D: 6-11 months, inclusive**	D1	1/8 dose	-----	30
		Placebo	-----	10
	D2	¼ dose	-----	30
		Placebo	-----	10
	D3	½ dose	-----	30
		Placebo	-----	10
	D4	¼ dose	2.5 µg	30
		Placebo	-----	10
	D5	¼ dose	5 µg	30
		Placebo	-----	10
			Total	200

*Presented study groups represent desired target sample size

Appendix 2. Flow Diagram for enrollment: 24-59 Month Olds

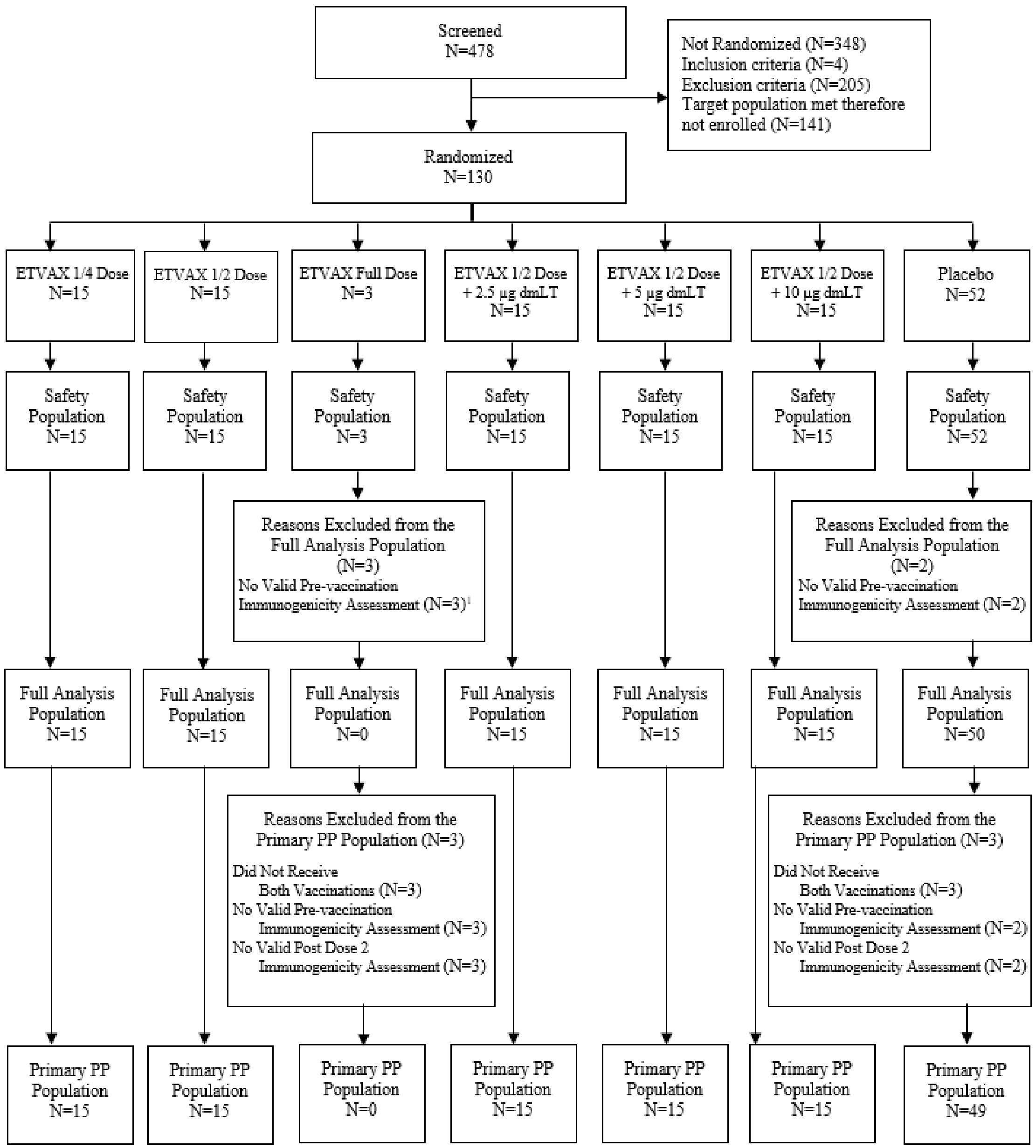

[1] Specimens were obtained but not analyzed.

Appendix 3. Flow Diagram for enrollment: 12-23 Month Olds

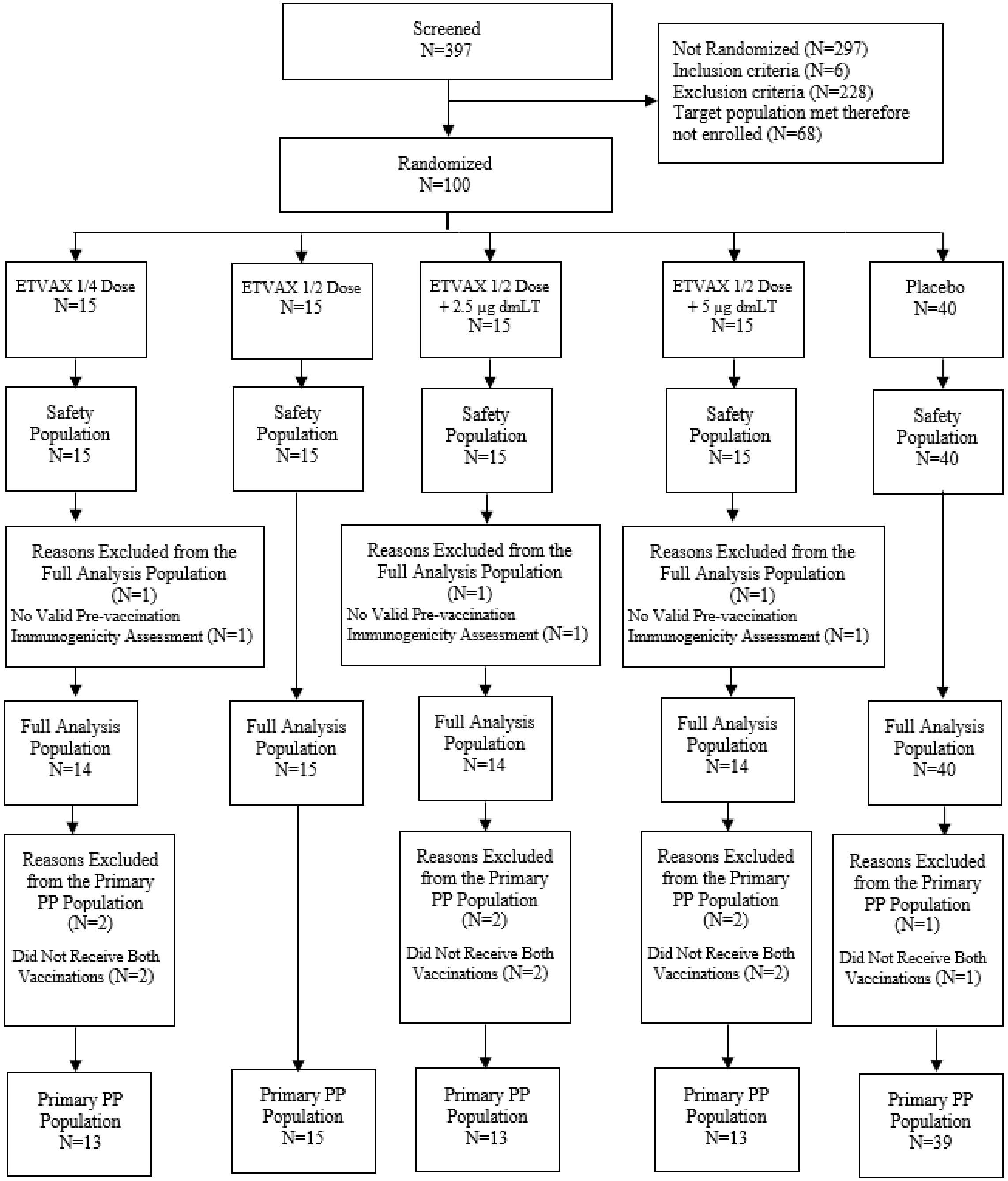

Appendix 4. Flow Diagram for enrollment: 6-11 Month Olds

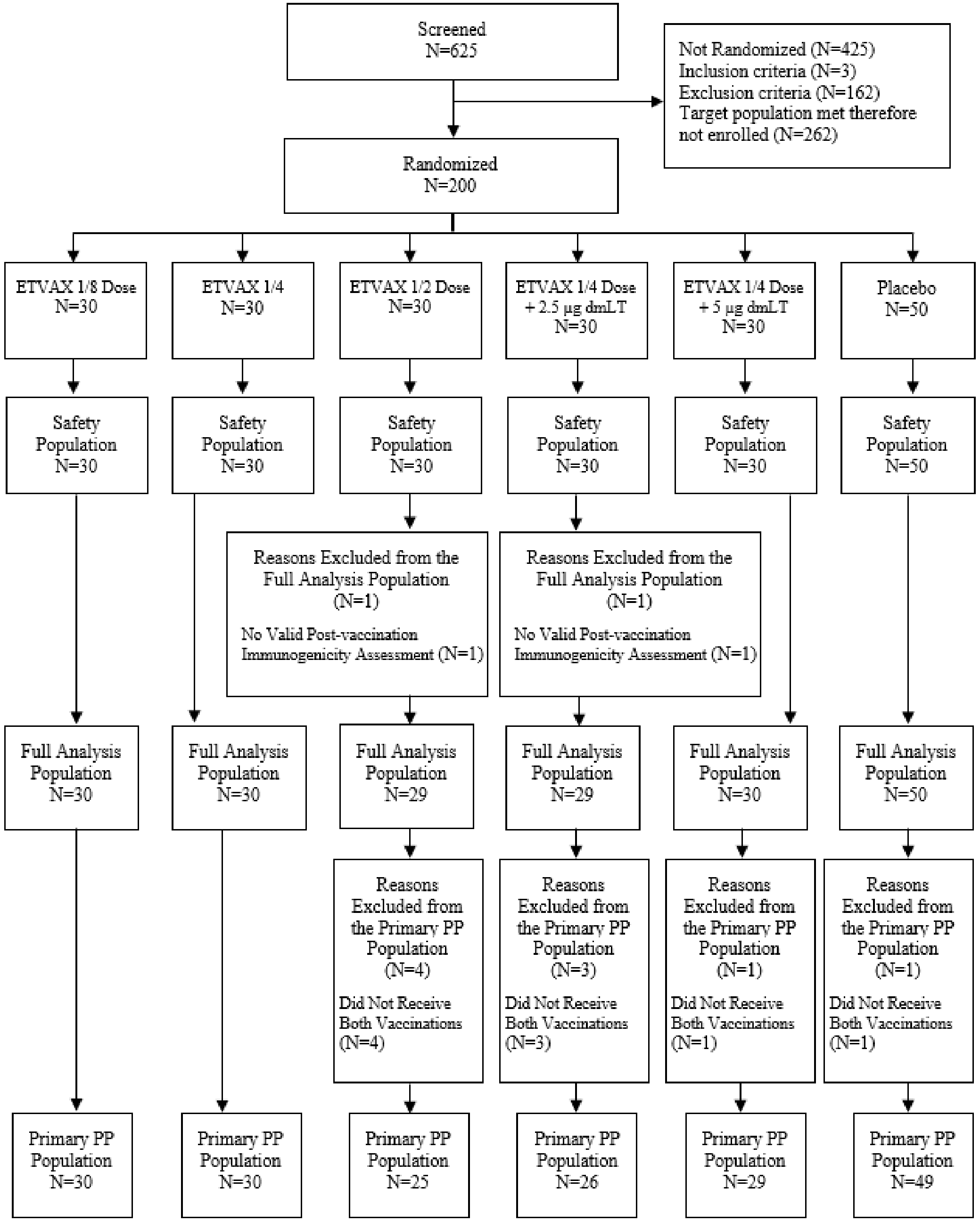

Appendix 5. Participant demographics

	Age (months)	Gender n (%)		Weight for Height n (%)	
	Mean (SD)	Female	Male	None	Mild
**Children 24-59 months**					
1/4 ETVAX (n=15)	49.1 (7.44)	10 (66.7)	5 (33.3)	7 (46.7)	8 (53.3)
1/2 ETVAX (n=15)	40.7 (8.17)	6 (40.0)	9 (60.0)	15 (100.0)	0 (0.0)
Full ETVAX (n=3)	50.7 (7.02)	2 (66.7)	1 (33.3)	0 (0.0)	3 (100.0)
1/2 ETVAX + 2.5 µg dmLT (n=15)	40.2 (10.36)	6 (40.0)	9 (60.0)	10 (66.7)	5 (33.3)
1/2 ETVAX + 5 µg dmLT (n=15)	43.5 (10.90)	7 (46.7)	8 (53.3)	10 (66.7)	5 (33.3)
1/2 ETVAX + 10 µg dmLT (n=15)	40.3 (8.80)	9 (60.0)	6 (40.0)	9 (60.0)	6 (40.0)
Placebo (n=52)	44.3 (9.29)	26 (50.0)	26 (50.0)	31 (59.6)	21 (40.4)
Total (n=130)	43.5 (9.46)	66 (50.8)	64 (49.2)	82 (63.1)	48 (36.9)
**Children 12-23 months**					
1/4 ETVAX (n=15)	15.9 (2.53)	6 (40.0)	9 (60.0)	9 (60.0)	6 (40.0)
1/2 ETVAX (n=15)	18.3 (3.58)	5 (33.3)	10 (66.7)	9 (60.0)	6 (40.0)
1/2 ETVAX + 2.5 µg dmLT (n=15)	19.1 (3.31)	7 (46.7)	8 (53.3)	7 (46.7)	8 (53.3)
1/2 ETVAX + 5 µg dmLT (n=15)	16.9 (3.18)	10 (66.7)	5 (33.3)	10 (66.7)	5 (33.3)
Placebo (n=40)	16.6 (3.25)	20 (50.0)	20 (50.0)	24 (60.0)	16 (40.0)
Total (n=100)	17.2 (3.32)	48 (48.0)	52 (52.0)	59 (59.0)	41 (41.0)
**Children 6-11 months**					
1/8 ETVAX (n=30)	8.2 (1.83)	14 (46.7)	16 (53.3)	22 (73.3)	8 (26.7)
1/4 ETVAX (n=30)	8.1 (1.63)	19 (63.3)	11 (36.7)	20 (66.7)	10 (33.3)
1/2 ETVAX (n=30)	8.2 (1.37)	16 (53.3)	14 (46.7)	22 (73.3)	8 (26.7)
1/4 ETVAX + 2.5 µg dmLT (n=30)	8.1 (1.80)	18 (60.0)	12 (40.0)	24 (80.0)	6 (20.0)
1/4 ETVAX + 5 µg dmLT (n=30)	7.6 (1.22)	16 (53.3)	14 (46.7)	26 (86.7)	4 (13.3)
Placebo (n=50)	7.8 (1.61)	23 (46.0)	27 (54.0)	42 (84.0)	8 (16.0)
Total (n=200)	8.0 (1.59)	106 (53.0)	94 (47.0)	156 (78.0)	44 (22.0)

Appendix 6. Multivariate analysis[1] of effect of age, vaccine dose and dmLT dose on vomiting

Odds of mild[2] or moderate[3] vomiting within 24 hours after receiving the first or second dose of ETVAX among children by age, vaccine dose, and dmLT dose

Variables	Vomiting<br>Present<br>n (%)	 Absent<br>n	Adjusted Odds Ratio[4]<br>(95% CI, p-value)	Trend Test
**Age (months)**				
**6 to 11**	27 (18.0)	123	5.44 (1.61 to 18.33, p=0.0063)	p=0.0035
**12 to 23**	13 (21.7)	47	3.98 (1.20 to 13.19, p=0.024)	
**24 to 59**	7 (9.3)	68	1	
**Vaccine Dose**				
**1/8**	2 (6.7)	28	0.18 (0.04 to 0.92, p=0.040)	p=0.0076
**1/4**	19 (15.8)	101	0.50 (0.22 to 1.15, p=0.10)	
**1/2**	26 (19.3)	109	1	
**dmLT (μg)**				
**0.0**	22 (14.7)	128	0.29 (0.05 to 1.53, p=0.14)	p=0.097
**2.5**	8 (13.3)	52	0.24 (0.04 to 1.41, p=0.11)	
**5.0**	14 (23.3)	46	0.49 (0.09 to 2.65, p=0.41)	
**10.0**	3 (20.0)	12	1	

[1] Odds Odds ratios were obtained by exponentiating model coefficients.
[2]Mild vomiting (n=42) is transient or intermittent vomiting with no or minimal interference with oral intake
[3] Moderate vomiting (n=5) is frequent episodes with no or mild dehydration
[4] Adjusted for all other variables in the model
Note: Model calibration was checked using the Hosmer and Lemeshow goodness-of-fit test. The p-value was 0.35 for the categorical model and 0.16 for the model including trend tests suggesting an adequate fit for both.

Appendix 7. Summary Laboratory Results by Study Day, and Treatment Group

Any Laboratory Parameter																				
					Mild/Grade 1				Moderate/Grade 2				Severe (Grade 3)				Severe (Grade 4)			
			None		(Low)		(High)		(Low)		(High)		(Low)		(High)		(Low)		(High)	
Time Point	Treatment Group	N	n	%	n	%	n	%	n	%	n	%	n	%	n	%	n	%	n	%
**24-59 Month Olds**																				
Baseline	Placebo	52	2	3.8	2	3.8	0	0.0	0	0.0	0	0.0	0	0.0	0	0.0	0	0.0	0	0.0
	ETVAX 1/4 Dose	15	2	13.3	0	0.0	0	0.0	0	0.0	0	0.0	0	0.0	0	0.0	0	0.0	0	0.0
	ETVAX 1/2 Dose	15	1	6.7	2	13.3	0	0.0	0	0.0	0	0.0	0	0.0	0	0.0	0	0.0	0	0.0
	ETVAX 1/2 Dose + 2.5 µg dmLT	15	1	6.7	0	0.0	0	0.0	0	0.0	0	0.0	0	0.0	0	0.0	0	0.0	0	0.0
	ETVAX 1/2 Dose + 5 µg dmLT	15	0	0.0	0	0.0	0	0.0	0	0.0	0	0.0	0	0.0	0	0.0	0	0.0	0	0.0
	ETVAX 1/2 Dose + 10 µg dmLT	15	3	20.0	0	0.0	0	0.0	0	0.0	0	0.0	0	0.0	0	0.0	0	0.0	0	0.0
Day 7	Placebo	52	8	15.4	3	5.8	2	3.8	1	1.9	0	0.0	0	0.0	0	0.0	0	0.0	0	0.0
	ETVAX 1/4 Dose	15	4	26.7	0	0.0	0	0.0	0	0.0	0	0.0	0	0.0	0	0.0	0	0.0	0	0.0
	ETVAX 1/2 Dose	15	0	0.0	3	20.0	0	0.0	0	0.0	0	0.0	0	0.0	0	0.0	0	0.0	0	0.0
	ETVAX 1/2 Dose + 2.5 µg dmLT	15	0	0.0	0	0.0	1	6.7	0	0.0	0	0.0	0	0.0	0	0.0	0	0.0	0	0.0
	ETVAX 1/2 Dose + 5 µg dmLT	15	0	0.0	1	6.7	0	0.0	0	0.0	0	0.0	0	0.0	0	0.0	0	0.0	0	0.0
	ETVAX 1/2 Dose + 10 µg dmLT	15	2	13.3	0	0.0	0	0.0	0	0.0	0	0.0	0	0.0	0	0.0	0	0.0	0	0.0
**12-23 Month Olds**																				
Baseline	**Placebo**	40	0	0.0	14	35.0	1	2.5	0	0.0	0	0.0	0	0.0	0	0.0	0	0.0	0	0.0
	ETVAX 1/4 Dose	15	0	0.0	8	53.3	1	6.7	0	0.0	0	0.0	0	0.0	0	0.0	0	0.0	0	0.0
	ETVAX 1/2 Dose	15	0	0.0	5	33.3	0	0.0	0	0.0	0	0.0	0	0.0	0	0.0	0	0.0	0	0.0
	ETVAX 1/2 Dose + 2.5 µg dmLT	15	0	0.0	7	46.7	0	0.0	0	0.0	0	0.0	0	0.0	0	0.0	0	0.0	0	0.0
	ETVAX 1/2 Dose + 5 µg dmLT	15	0	0.0	6	40.0	0	0.0	0	0.0	0	0.0	0	0.0	0	0.0	0	0.0	0	0.0
Day 7	Placebo	40	0	0.0	15	37.5	2	5.0	1	2.5	3	7.5	0	0.0	0	0.0	0	0.0	0	0.0
	ETVAX 1/4 Dose	14	0	0.0	6	42.9	2	14.3	2	14.3	0	0.0	0	0.0	1	7.1	0	0.0	0	0.0
	ETVAX 1/2 Dose	15	0	0.0	4	26.7	0	0.0	0	0.0	0	0.0	0	0.0	0	0.0	0	0.0	0	0.0
	ETVAX 1/2 Dose + 2.5 µg dmLT	14	0	0.0	8	57.1	0	0.0	0	0.0	1	7.1	0	0.0	0	0.0	0	0.0	0	0.0
	ETVAX 1/2 Dose + 5 µg dmLT	14	0	0.0	6	42.9	1	7.1	1	7.1	0	0.0	0	0.0	0	0.0	0	0.0	0	0.0
**6-11 Month Olds**																				
Baseline	Placebo	50	0	0.0	3	6.0	0	0.0	0	0.0	0	0.0	0	0.0	0	0.0	0	0.0	0	0.0
	ETVAX 1/8 Dose	30	0	0.0	3	10.0	1	3.3	0	0.0	0	0.0	0	0.0	0	0.0	0	0.0	0	0.0
	ETVAX 1/4 Dose	30	0	0.0	7	23.3	0	0.0	0	0.0	0	0.0	0	0.0	0	0.0	0	0.0	0	0.0
	ETVAX 1/2 Dose	30	0	0.0	2	6.7	0	0.0	0	0.0	0	0.0	0	0.0	0	0.0	0	0.0	0	0.0
	ETVAX 1/4 Dose + 2.5 µg dmLT	30	0	0.0	1	3.3	0	0.0	0	0.0	0	0.0	0	0.0	0	0.0	0	0.0	0	0.0
	ETVAX 1/4 Dose + 5 µg dmLT	30	0	0.0	0	0.0	0	0.0	0	0.0	0	0.0	0	0.0	0	0.0	0	0.0	0	0.0
Day 7	Placebo	50	0	0.0	12	24.0	2	4.0	0	0.0	1	2.0	0	0.0	0	0.0	0	0.0	0	0.0
	ETVAX 1/8 Dose	30	0	0.0	4	13.3	3	10.0	0	0.0	0	0.0	0	0.0	0	0.0	0	0.0	0	0.0
	ETVAX 1/4 Dose	30	0	0.0	7	23.3	7	23.3	0	0.0	0	0.0	0	0.0	0	0.0	0	0.0	0	0.0
	ETVAX 1/2 Dose	29	0	0.0	6	20.7	3	10.3	0	0.0	0	0.0	0	0.0	0	0.0	0	0.0	0	0.0
	ETVAX 1/4 Dose + 2.5 µg dmLT	29	0	0.0	6	20.7	2	6.9	0	0.0	0	0.0	0	0.0	0	0.0	0	0.0	0	0.0
	ETVAX 1/4 Dose + 5 µg dmLT	30	0	0.0	5	16.7	4	13.3	0	0.0	0	0.0	0	0.0	0	0.0	0	0.0	0	0.0

Day 7 was summarized using Visit 3 data. For some participants, Day 7 (or Visit 3) occurred on Day 6 or Day 8. These cases were included in the Day 7 summary.
N = Number of participants in the Safety population.
Note: Laboratory toxicity grading was carried out according to the "Division of AIDS Table for Grading the Severity of Adult and Pediatric Adverse Events, 2009, of the US National Institutes of Health

Appendix 8. Hemoglobin Laboratory Results by Study Day, and Treatment Group

Hemoglobin (g/dL)																					
					Mild/Grade 1				Moderate/Grade 2				Severe (Grade 3)				Severe (Grade 4)				
			None		(Low)		(High)		(Low)		(High)		(Low)		(High)		(Low)		(High)		
Time Point	Treatment Group	N	n	%	n	%	n	%	n	%	n	%	n	%	n	%	n	%	n	%	
**24-59 Month Olds***																					
Baseline	Placebo	52	18	34.6	2	3.8	0	0.0	0	0.0	0	0.0	0	0.0	0	0.0	0	0.0	0	0.0	
	ETVAX 1/4 Dose	15	6	40.0	0	0.0	0	0.0	0	0.0	0	0.0	0	0.0	0	0.0	0	0.0	0	0.0	
	ETVAX 1/2 Dose	15	7	46.7	2	13.3	0	0.0	0	0.0	0	0.0	0	0.0	0	0.0	0	0.0	0	0.0	
	ETVAX 1/2 Dose + 2.5 µg dmLT	15	8	53.3	0	0.0	0	0.0	0	0.0	0	0.0	0	0.0	0	0.0	0	0.0	0	0.0	
	ETVAX 1/2 Dose + 5 µg dmLT	15	2	13.3	0	0.0	0	0.0	0	0.0	0	0.0	0	0.0	0	0.0	0	0.0	0	0.0	
	ETVAX 1/2 Dose + 10 µg dmLT	15	9	60.0	0	0.0	0	0.0	0	0.0	0	0.0	0	0.0	0	0.0	0	0.0	0	0.0	
Day 7	Placebo	52	19	36.5	3	5.8	0	0.0	1	1.9	0	0.0	0	0.0	0	0.0	0	0.0	0	0.0	
	ETVAX 1/4 Dose	15	9	60.0	0	0.0	0	0.0	0	0.0	0	0.0	0	0.0	0	0.0	0	0.0	0	0.0	
	ETVAX 1/2 Dose	15	6	40.0	3	20.0	0	0.0	0	0.0	0	0.0	0	0.0	0	0.0	0	0.0	0	0.0	
	ETVAX 1/2 Dose + 2.5 µg dmLT	15	6	40.0	0	0.0	0	0.0	0	0.0	0	0.0	0	0.0	0	0.0	0	0.0	0	0.0	
	ETVAX 1/2 Dose + 5 µg dmLT	15	5	33.3	1	6.7	0	0.0	0	0.0	0	0.0	0	0.0	0	0.0	0	0.0	0	0.0	
	ETVAX 1/2 Dose + 10 µg dmLT	15	9	60.0	0	0.0	0	0.0	0	0.0	0	0.0	0	0.0	0	0.0	0	0.0	0	0.0	
**12-23 Month Olds***																					
Baseline	Placebo	40	8	20.0	14	35.0	0	0.0	0	0.0	0	0.0	0	0.0	0	0.0	0	0.0	0	0.0	
	ETVAX 1/4 Dose	15	2	13.3	8	53.3	0	0.0	0	0.0	0	0.0	0	0.0	0	0.0	0	0.0	0	0.0	
	ETVAX 1/2 Dose	15	1	6.7	5	33.3	0	0.0	0	0.0	0	0.0	0	0.0	0	0.0	0	0.0	0	0.0	
	ETVAX 1/2 Dose + 2.5 µg dmLT	15	3	20.0	7	46.7	0	0.0	0	0.0	0	0.0	0	0.0	0	0.0	0	0.0	0	0.0	
	ETVAX 1/2 Dose + 5 µg dmLT	15	3	20.0	6	40.0	0	0.0	0	0.0	0	0.0	0	0.0	0	0.0	0	0.0	0	0.0	
Day 7	Placebo	40	9	22.5	15	37.5	0	0.0	1	2.5	0	0.0	0	0.0	0	0.0	0	0.0	0	0.0	
	ETVAX 1/4 Dose	14	2	14.3	6	42.9	0	0.0	2	14.3	0	0.0	0	0.0	0	0.0	0	0.0	0	0.0	
	ETVAX 1/2 Dose	15	2	13.3	4	26.7	0	0.0	0	0.0	0	0.0	0	0.0	0	0.0	0	0.0	0	0.0	
	ETVAX 1/2 Dose + 2.5 µg dmLT	14	3	21.4	8	57.1	0	0.0	0	0.0	0	0.0	0	0.0	0	0.0	0	0.0	0	0.0	
	ETVAX 1/2 Dose + 5 µg dmLT	14	2	14.3	6	42.9	0	0.0	1	7.1	0	0.0	0	0.0	0	0.0	0	0.0	0	0.0	
**6-11 Month Olds****																					
Baseline	Placebo	50	1	2.0	3	6.0	0	0.0	0	0.0	0	0.0	0	0.0	0	0.0	0	0.0	0	0.0	
	ETVAX 1/8 Dose	30	4	13.3	3	10.0	0	0.0	0	0.0	0	0.0	0	0.0	0	0.0	0	0.0	0	0.0	
	ETVAX 1/4 Dose	30	0	0.0	7	23.3	0	0.0	0	0.0	0	0.0	0	0.0	0	0.0	0	0.0	0	0.0	
	ETVAX 1/2 Dose	30	2	6.7	2	6.7	0	0.0	0	0.0	0	0.0	0	0.0	0	0.0	0	0.0	0	0.0	
	ETVAX 1/4 Dose + 2.5 µg dmLT	30	3	10.0	1	3.3	0	0.0	0	0.0	0	0.0	0	0.0	0	0.0	0	0.0	0	0.0	
	ETVAX 1/4 Dose + 5 µg dmLT	30	5	16.7	0	0.0	0	0.0	0	0.0	0	0.0	0	0.0	0	0.0	0	0.0	0	0.0	
Day 7	Placebo	50	1	2.0	12	24.0	0	0.0	0	0.0	0	0.0	0	0.0	0	0.0	0	0.0	0	0.0	
	ETVAX 1/8 Dose	30	3	10.0	4	13.3	0	0.0	0	0.0	0	0.0	0	0.0	0	0.0	0	0.0	0	0.0	
	ETVAX 1/4 Dose	30	1	3.3	7	23.3	0	0.0	0	0.0	0	0.0	0	0.0	0	0.0	0	0.0	0	0.0	
	ETVAX 1/2 Dose	29	1	3.4	6	20.7	0	0.0	0	0.0	0	0.0	0	0.0	0	0.0	0	0.0	0	0.0	
	ETVAX 1/4 Dose + 2.5 µg dmLT	29	2	6.9	6	20.7	0	0.0	0	0.0	0	0.0	0	0.0	0	0.0	0	0.0	0	0.0	
	ETVAX 1/4 Dose + 5 µg dmLT	30	5	16.7	5	16.7	0	0.0	0	0.0	0	0.0	0	0.0	0	0.0	0	0.0	0	0.0	

Day 7 was summarized using Visit 3 data. For some participants, Day 7 (or Visit 3) occurred on Day 6 or Day 8. These cases were included in the Day 7 summary.
N = Number of participants in the Safety population.
*Hemoglobin laboratory toxicity grading 12-59 months: Mild/Grade 1 = 10.0-10.9; Moderate/Grade 2 = 7.0-9.9; Severe/Grade 3 = <7.0; Severe/Grade 4 = Cardiac failure secondary to anaemia
**Hemoglobin laboratory toxicity grading 6-11 months: Mild/Grade 1 = 0.0-9.9; Moderate/Grade 2 = 7.0-8.9; Severe/Grade 3 = <7.0; Severe/Grade 4 = Cardiac failure secondary to anaemia

Appendix 9. White Blood Cell (WBC) Laboratory Results by Study Day, and Treatment Group

WBC (x10/L)																				
					Mild/Grade 1				Moderate/Grade 2				Severe (Grade 3)				Severe (Grade 4)			
			None		(Low)		(High)		(Low)		(High)		(Low)		(High)		(Low)		(High)	
Time Point	Treatment Group	N	n	%	n	%	n	%	n	%	n	%	n	%	n	%	n	%	n	%
**24-59 Month Olds**																				
Baseline	Placebo	52	49	94.2	0	0.0	0	0.0	0	0.0	0	0.0	0	0.0	0	0.0	0	0.0	0	0.0
	ETVAX 1/4 Dose	15	15	100.0	0	0.0	0	0.0	0	0.0	0	0.0	0	0.0	0	0.0	0	0.0	0	0.0
	ETVAX 1/2 Dose	15	15	100.0	0	0.0	0	0.0	0	0.0	0	0.0	0	0.0	0	0.0	0	0.0	0	0.0
	ETVAX 1/2 Dose + 2.5 µg dmLT	15	14	93.3	0	0.0	0	0.0	0	0.0	0	0.0	0	0.0	0	0.0	0	0.0	0	0.0
	ETVAX 1/2 Dose + 5 µg dmLT	15	14	93.3	0	0.0	0	0.0	0	0.0	0	0.0	0	0.0	0	0.0	0	0.0	0	0.0
	ETVAX 1/2 Dose + 10 µg dmLT	15	15	100.0	0	0.0	0	0.0	0	0.0	0	0.0	0	0.0	0	0.0	0	0.0	0	0.0
Day 7	Placebo	52	50	96.2	0	0.0	2	3.8	0	0.0	0	0.0	0	0.0	0	0.0	0	0.0	0	0.0
	ETVAX 1/4 Dose	15	15	100.0	0	0.0	0	0.0	0	0.0	0	0.0	0	0.0	0	0.0	0	0.0	0	0.0
	ETVAX 1/2 Dose	15	15	100.0	0	0.0	0	0.0	0	0.0	0	0.0	0	0.0	0	0.0	0	0.0	0	0.0
	ETVAX 1/2 Dose + 2.5 µg dmLT	15	12	80.0	0	0.0	1	6.7	0	0.0	0	0.0	0	0.0	0	0.0	0	0.0	0	0.0
	ETVAX 1/2 Dose + 5 µg dmLT	15	14	93.3	0	0.0	0	0.0	0	0.0	0	0.0	0	0.0	0	0.0	0	0.0	0	0.0
	ETVAX 1/2 Dose + 10 µg dmLT	15	15	100.0	0	0.0	0	0.0	0	0.0	0	0.0	0	0.0	0	0.0	0	0.0	0	0.0
**12-23 Month Olds**																				
Baseline	Placebo	40	39	97.5	0	0.0	1	2.5	0	0.0	0	0.0	0	0.0	0	0.0	0	0.0	0	0.0
	ETVAX 1/4 Dose	15	14	93.3	0	0.0	1	6.7	0	0.0	0	0.0	0	0.0	0	0.0	0	0.0	0	0.0
	ETVAX 1/2 Dose	15	15	100.0	0	0.0	0	0.0	0	0.0	0	0.0	0	0.0	0	0.0	0	0.0	0	0.0
	ETVAX 1/2 Dose + 2.5 µg dmLT	15	15	100.0	0	0.0	0	0.0	0	0.0	0	0.0	0	0.0	0	0.0	0	0.0	0	0.0
	ETVAX 1/2 Dose + 5 µg dmLT	15	15	100.0	0	0.0	0	0.0	0	0.0	0	0.0	0	0.0	0	0.0	0	0.0	0	0.0
Day 7	Placebo	40	34	85.0	0	0.0	2	5.0	0	0.0	2	5.0	0	0.0	0	0.0	0	0.0	0	0.0
	ETVAX 1/4 Dose	14	11	78.6	0	0.0	2	14.3	0	0.0	0	0.0	0	0.0	1	7.1	0	0.0	0	0.0
	ETVAX 1/2 Dose	15	15	100.0	0	0.0	0	0.0	0	0.0	0	0.0	0	0.0	0	0.0	0	0.0	0	0.0
	ETVAX 1/2 Dose + 2.5 µg dmLT	14	13	92.9	0	0.0	0	0.0	0	0.0	1	7.1	0	0.0	0	0.0	0	0.0	0	0.0
	ETVAX 1/2 Dose + 5 µg dmLT	14	13	92.9	0	0.0	1	7.1	0	0.0	0	0.0	0	0.0	0	0.0	0	0.0	0	0.0
**6-11 Month Olds**																				
Baseline	Placebo	50	50	100.0	0	0.0	0	0.0	0	0.0	0	0.0	0	0.0	0	0.0	0	0.0	0	0.0
	ETVAX 1/8 Dose	30	28	93.3	0	0.0	1	3.3	0	0.0	0	0.0	0	0.0	0	0.0	0	0.0	0	0.0
	ETVAX 1/4 Dose	30	30	100.0	0	0.0	0	0.0	0	0.0	0	0.0	0	0.0	0	0.0	0	0.0	0	0.0
	ETVAX 1/2 Dose	30	30	100.0	0	0.0	0	0.0	0	0.0	0	0.0	0	0.0	0	0.0	0	0.0	0	0.0
	ETVAX 1/4 Dose + 2.5 µg dmLT	30	30	100.0	0	0.0	0	0.0	0	0.0	0	0.0	0	0.0	0	0.0	0	0.0	0	0.0
	ETVAX 1/4 Dose + 5 µg dmLT	30	30	100.0	0	0.0	0	0.0	0	0.0	0	0.0	0	0.0	0	0.0	0	0.0	0	0.0
Day 7	Placebo	50	48	96.0	0	0.0	2	4.0	0	0.0	0	0.0	0	0.0	0	0.0	0	0.0	0	0.0
	ETVAX 1/8 Dose	30	28	93.3	0	0.0	2	6.7	0	0.0	0	0.0	0	0.0	0	0.0	0	0.0	0	0.0
	ETVAX 1/4 Dose	30	23	76.7	0	0.0	7	23.3	0	0.0	0	0.0	0	0.0	0	0.0	0	0.0	0	0.0
	ETVAX 1/2 Dose	29	25	86.2	0	0.0	3	10.3	0	0.0	0	0.0	0	0.0	0	0.0	0	0.0	0	0.0
	ETVAX 1/4 Dose + 2.5 µg dmLT	29	27	93.1	0	0.0	2	6.9	0	0.0	0	0.0	0	0.0	0	0.0	0	0.0	0	0.0
	ETVAX 1/4 Dose + 5 µg dmLT	30	27	90.0	0	0.0	2	6.7	0	0.0	0	0.0	0	0.0	0	0.0	0	0.0	0	0.0

Day 7 was summarized using Visit 3 data. For some participants, Day 7 (or Visit 3) occurred on Day 6 or Day 8. These cases were included in the Day 7 summary.
N = Number of participants in the Safety population.
*WBC laboratory toxicity grading 12-59 months: Decreased: Mild/Grade 1 = 2,000-3,999; Moderate/Grade 2 = 1,500-1,999; Severe/Grade 3 = 1,000-1,499; Severe/Grade 4 = <1,000
Increased: Mild/Grade 1 = 11,000-15,000; Moderate/Grade 2 = 15,001-20,000; Severe/Grade 3 = 20,001-25,000; Severe/Grade 4 = >25,000
**WBC laboratory toxicity grading 6-11 months: Decreased: Mild/Grade 1 = 2,000-2,500; Moderate/Grade 2 = 1,500-1,999; Severe/Grade 3 = 1,000-1,499; Severe/Grade 4 = <1,000
Increased: Mild/Grade 1 = 17,501-22,750; Moderate/Grade 2 = 22,751-29,750; Severe/Grade 3 = 29,751-38,500; Severe/Grade 4 = >38,500

Appendix 10. ALS IgA responses in the different B cohorts of children 24-59 months

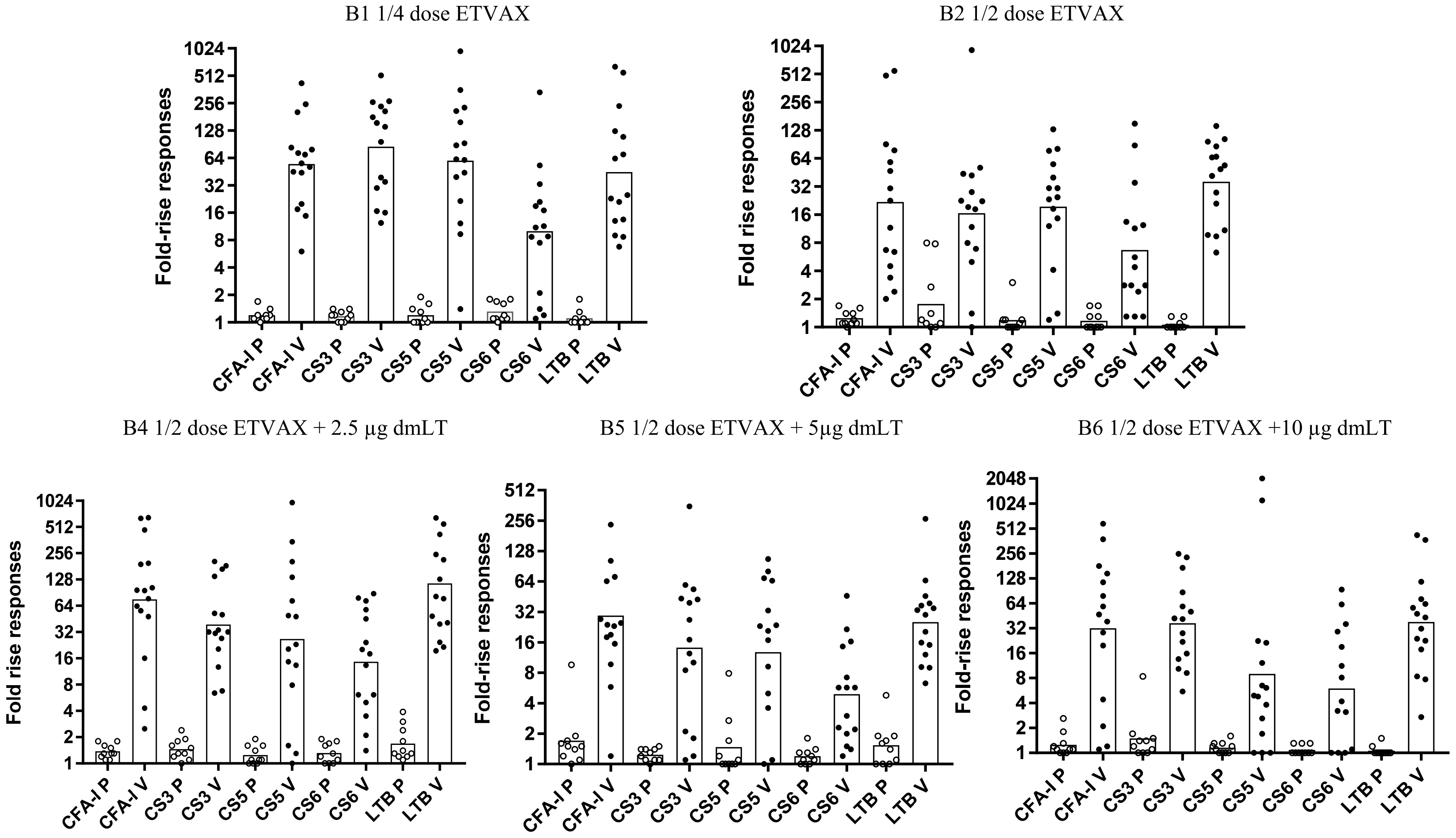

Fold-rise (geometric mean and range) ALS IgA responses from baseline to day of peak response – either Day 7 after the first dose or Day 5 after the second dose – against ETVAX antigens in Vaccinees (V) and Placebo (P) recipients 24-59 months of age given 1/4 and 1/2 dose of ETVAX and ½ dose of ETVAX + different doses of dmLT; statistical differences for all comparisons V vs P for all antigens p≤0.01 (pairwise comparisons based on 2-sample t-test with or wihout Holm's-Bonferroni adjustment for multiple antigens).

Appendix 11. Numbers and frequencies (%) of responders in ALS IgA in the B cohort and C cohort children

	CFA/I	CS3	CS5	CS6	LTB
Cohort B (B1-B6): 24-59 months;					
V (n=75)	73 (97%)	70 (93%)	64 (85%)	61 (81%)	75 (100%)
P (n=50)	2 (4%)	5 (10%)	3 (6%)	1 (2%)	4 (8%)
Cohort C (C1-C4): 12-23 months					
V (n=57)	42 (74%)	45 (79%)	32 (56%)	29 (51%)	51 (95%)
P (n=40)	3 (7.5%)	2 (5%)	1 (2.5%)	6 (16%)	4 (10%)

Responder frequencies (%; ≥2-fold rises) of ALS IgA responses from baseline to day of peak response – either Day 7 after the first dose or Day 5 after the second dose – against ETVAX antigens in Vaccinees (V) and Placebo (P) recipients given different doses of ETVAX ± dmLT. Statistical differences between responder frequencies in V and P, $p<0.0001$ for all antigens in both age groups (Fisher's Exact 2-tailed Test; $p<0.0005$ for all antigens in both groups with adjustments).

Appendix 12. ALS IgA responses in the different C cohorts of children 12-23 months

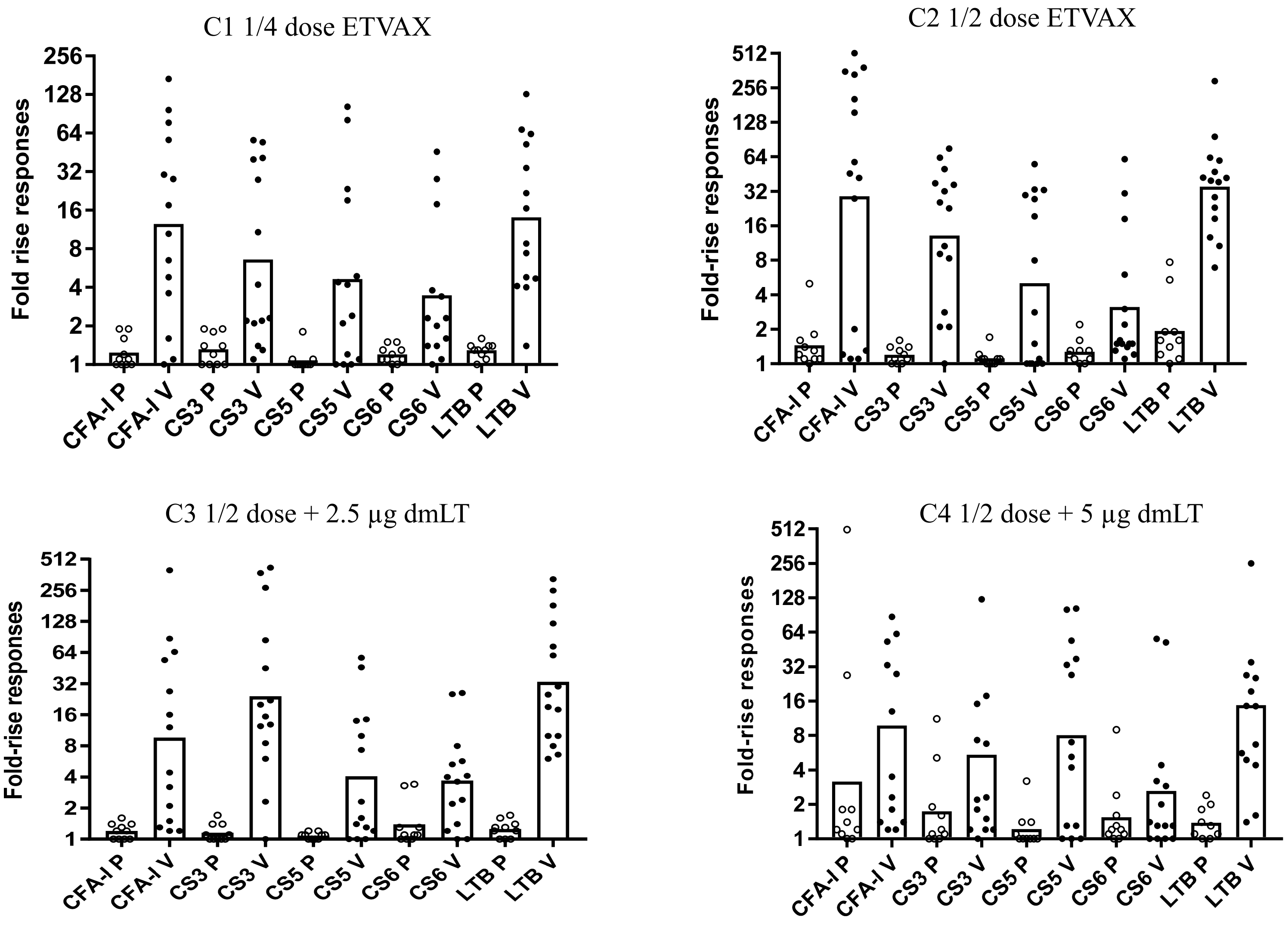

Fold-rise (geometric mean and range) ALS IgA responses from baseline to day of peak response – either Day 7 after the first dose or Day 5 after the second dose – against ETVAX antigens in Vaccinees (V) and Placebo (P) recipients 12-23 months; statistical differences V vs P, p≤0.04 to p≤ 0.0001 for all antigens in cohorts C1-C3, in cohort C4 only significant for C5 (≤0.004) and LTB (p=0.0005) (2-sample t-test); with Holm's-Bonferroni adjustment for multiple antigens p≤ 0.04 for all antigens in cohorts C1-C3 and CS5 and LTB in cohort 4.

Appendix 13. Number and frequencies (%) of responders in ALS IgA in 6-11 months old children given ETVAX or placebo (all D cohorts)

	Total n	CFA/I	CS3	CS5	CS6	LTB
Vaccine (V)	144	54 (38%)	58 (40%)	26 (18%)	21 (15%)	107 (74%)
Placebo (P)	50	8 (16%)	4 (8%)	7 (14%)	2 (4%)	14 (28%)
P-value V vs P*<br>P value V vs P**		0.005<br>0.02	<0.0001<br><0.0005	ns<br>ns	ns<br>ns	<0.0001<br><0.0005

* Fisher's exact test,
** Fisher's exact test with Holm's Bonferroni adjustment
ns Not significant

Appendix 14.

Lactoferrin response frequencies (%) and GMFR in infants responding to CFA/I and LTB, respectively in representative D cohorts and treatment groups

		CFA/1		Lactoferrin[e]		LTB		Lactoferrin[e]	
Cohort[a]	Group	Responders[b]	GMFR[c]	Responders[d]	GMFR[c]	Responders[b]	GMFR[c]	Responders[d]	GMFR[c]
D1	Vaccine	13/24 (54.1)	9.0	1/13 (7.7)	4.5	13/24 (54.1)	15.0	1/13 (7.7)	2.0
D1	Placebo	5/10 (50.0)	4.0	2/5 (40.0)	2.5	5/10 (50.0)	3.9	2/5 (40.0)	2.5
D4	Vaccine	14/25 (56.0)	15.0	4/14 (28.6)	2.7	16/25 (64.0)	41.0	4/16 (25.0)	2.7
D4	Placebo	2/8 (25.0)	2.7	0/2 (0.0)	1.0	2/8 (25.0)	90.0[f]	2/2 (100)	2.4

a) Cohort D1 (1/8 dose ETVAX or placebo); Cohort D4 (1/4 ETVAX + 2.5ug dmLT or placebo)
b) 2-fold or greater rise from baseline/persons tested (percent responders)
c) Geometric mean fold rise from baseline in CFA/I or LTB responders only
d) 2-fold or greater rise from baseline/persons tested (percent responders) among CFA/1 or LTB responders; maximal responses for each individual
e) Among infants mounting faecal IgA responses to either CFA/I or LTB
f) The two high anti-LTB responders in the placebo group were likely the result of asymptomatic infections with either an ETEC or vibrio

Appendix 15. Numbers and frequencies (%) of responders in ALS IgA and/or faecal SIgA against ETVAX antigens in D cohort vaccinees and placebo recipients 6-11 months of age

	CFA/I	CS3	CS5	CS6	LTB
Vaccinees* n=144	86 (60%)	89 (62%)	70 (49%)	70 (49%)	116 (81%)
Placebo n=50	17 (34%)	14 (28%)	14 (28%)	15 (30%)	26 (52%)
Stat. diff V/P Fisher's exact test with corrections**	P=0.002<br>P=0.006	P<0.0001<br>P<0.0005	P=0.013<br>P=0.026	P=0.02<br>P=0.02	P=0.0001<br>P=0.0004

*All given vaccine alone and vaccine + dmLT
** Holm's Bonferroni adjustments for multiple antigens

Appendix 16. Plasma IgA and IgG (LTB only) responses in the different B cohorts of children 24-59 months

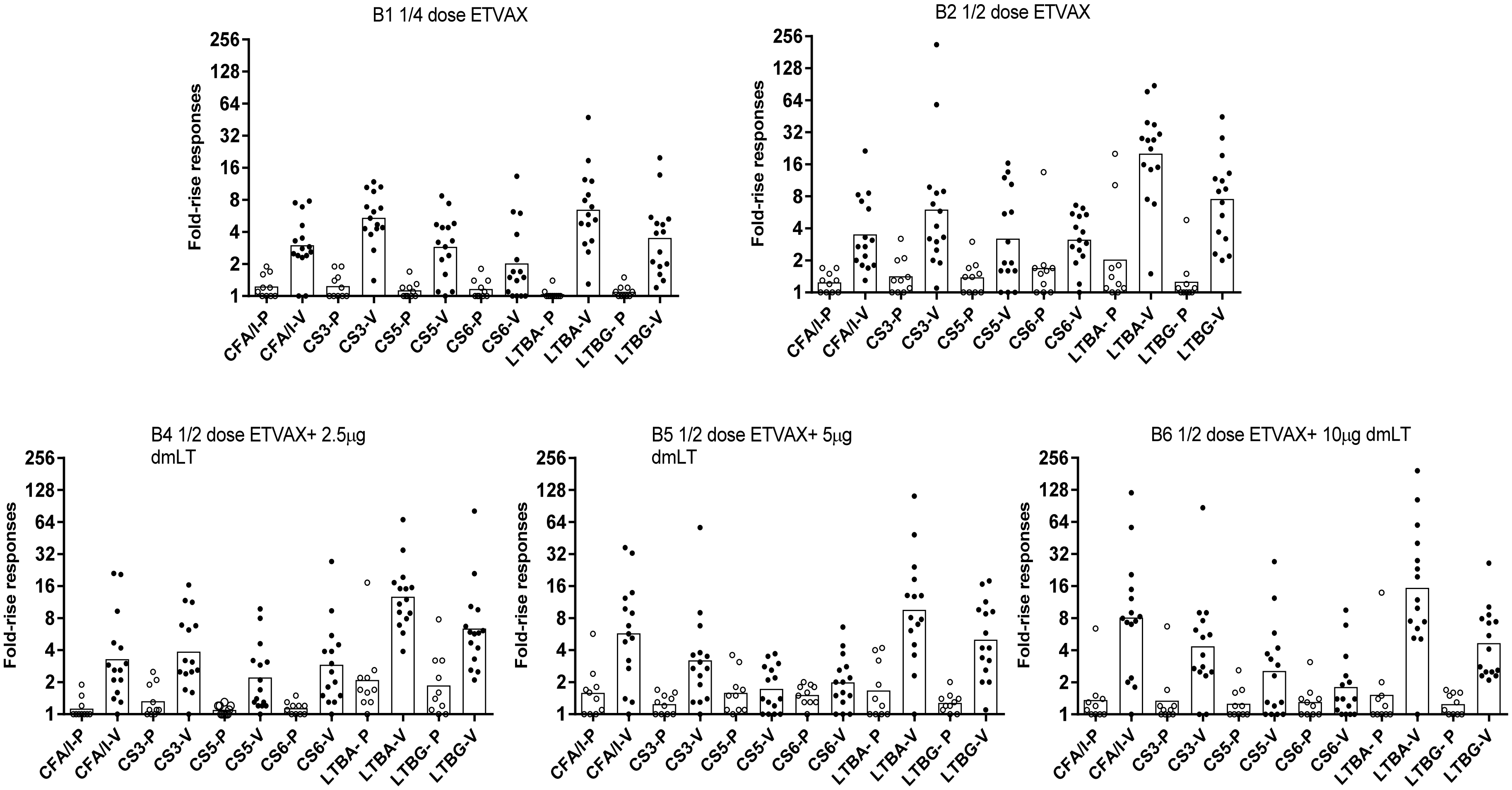

Fold-rise (geometric mean and range) plasma antibody responses from baseline to day of peak response – either Day 7 after the first dose or Day 5 after the second dose – against ETVAX antigens in Vaccinees (V) and Placebo (P) recipients 24-59 months of age; statistical differences V vs P $p \leq 0.05$ for all antigens except for C5 and CS6 in cohorts B5 and CS6 in cohort B6 (2-sample t-test); with Holm's-Bonferroni adjustment for multiple antigens $p \leq 0.05$ for all antigens in all cohorts except CS5 and CS6 in cohorts B2, B5 and B6.

Appendix 17. Plasma IgA antibody responses in the different C cohorts of children 12-23 months

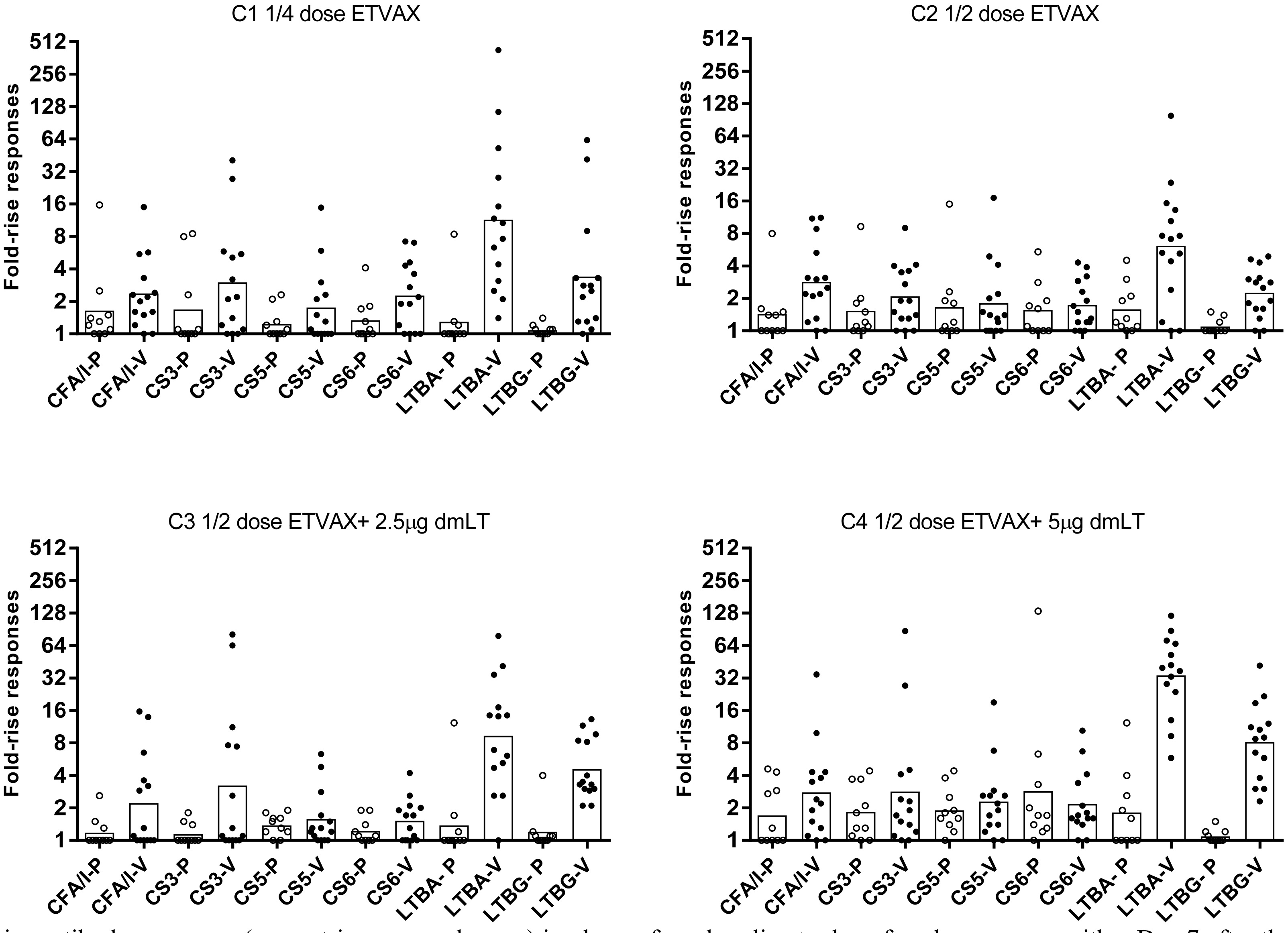

Fold-rise antibody responses (geometric mean and range) in plasma from baseline to day of peak response – either Day 7 after the first dose or Day 5 after the second dose – against vaccine antigens in 12-23 months old children (IgA responses to all antigens and IgG also to LTB). Statistical differences V vs P, $p<0.05$ for CFA/I (C2) and $p \leq 0.01$ for LTB IgA and IgG in all cohorts (2-sample t-test); with Holm's-Bonferroni adjustment for multiple antigens $p \leq 0.02$ for LTB IgA and IgG except LTB IgG C1 (ns)

Appendix 18. Plasma IgA antibody responses in the different D cohorts of infants 6-11 months

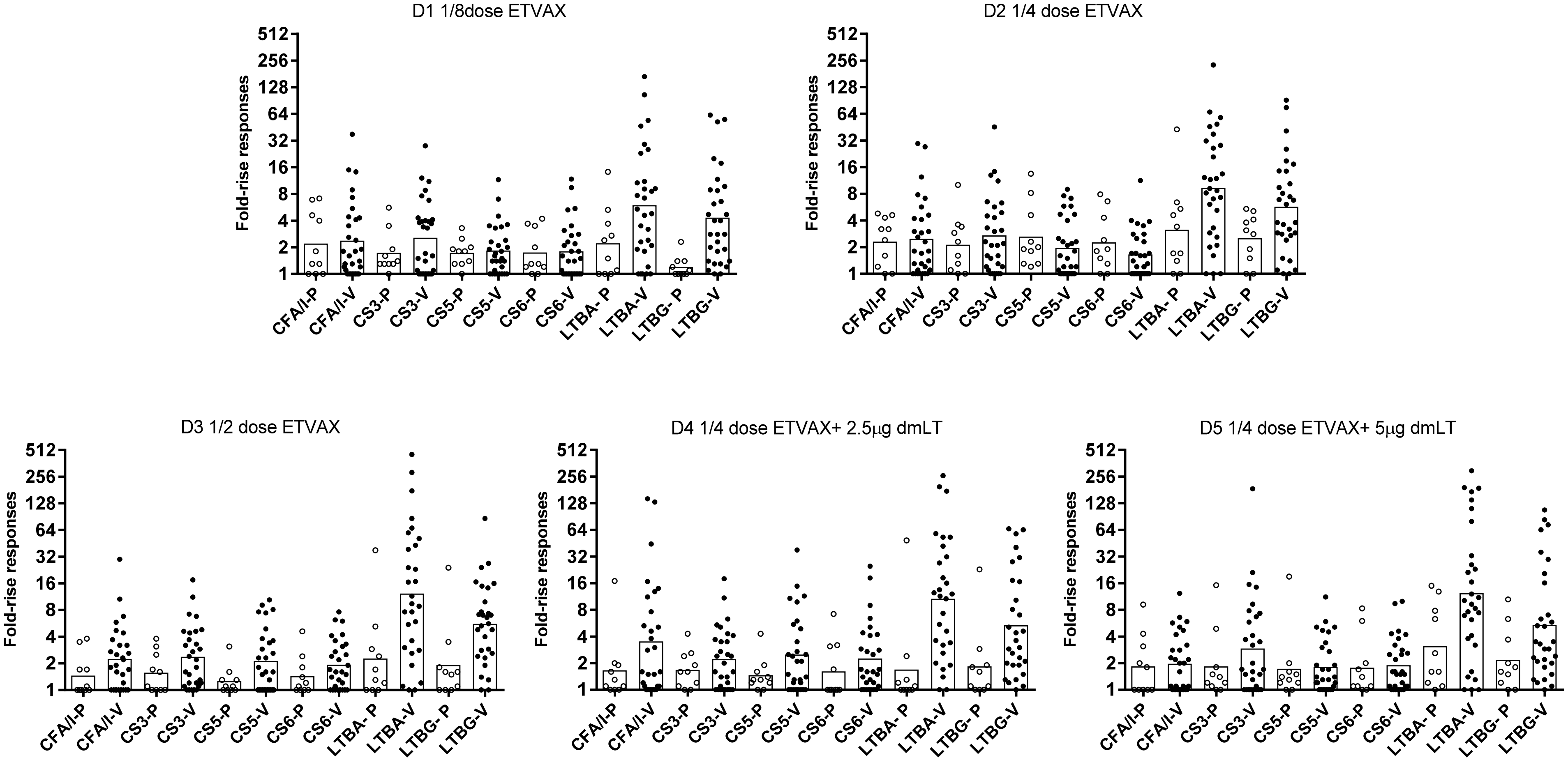

Fold-rise antibody responses (geometric mean and range) in plasma from baseline to day of peak response – either Day 7 after the first dose or Day 5 after the second dose – against vaccine antigens in 6-11 months old children given ETVAX ± dmLT (IgA responses to all antigens and IgG also to LTB). Statistical differences V vs P, $p \leq 0.05$ for LTB IgA in all cohorts and for LTB IgG $p \leq 0.05$ (cohorts D1, D3 and D4) (2-sample t-test); with Holm's-Bonferroni adjustment for multiple antigens $p \leq 0.05$ for LTB IgA in cohorts D3 and D4 and for LTB IgG in cohorts D1 and D3

Appendix 19. Number and frequencies (%) of plasma IgA responders in the different Cohorts B, C and D

	CFA/I	CS3	CS5	CS6	LTB
Cohort B: 24-59 months					
V(n=75)	62 (81%)	40 (83%)	39 (53%)	39 (52%)	71 (95%)
P(n=50)	4 (8%)	4 (8%)	4 (8%)	3 (6%)	11 (22%)
Cohort C: 12-23 months					
V (n=57)	33 (58%)	26 (46%)	20 (35%)	21 (37%)	51 (89%)
P (n=40)	8 (20%)	9 (23%)	7 (18%)	7(18%)	8 (20%)
Cohort D: 6-11 months					
V(n=144)	67 (46%)	74 (51%)	57 (41%)	53 (39%)	116 (81%)
P (n=50)	18 (36%)	15 (30%)	12 (24%)	16 (32%)	22 (44%)

Responder frequencies ($\geq$ 2-fold rises) of plasma IgA responses from baseline to day of peak response – either Day 7 after the first dose or Day 5 after the second dose – against ETVAX antigens in Vaccinees (V) and Placebo (P) recipients of all cohorts. Statistical differences in responder frequencies V vs P: (a) 24-59 months $p<0.0001$ for all antigens; after adjustments $p<0.0005$, (b) 12-23 months of age, $p\leq0.02$ for all antigens except CS5 (ns); with adjustments $p\leq0.008$ for CFA/I and LTB (c) 6-11 months of age, $p=0.014$ for CS3, $p=0.04$ for CS5, $p<0.0001$ for LTB; with adjustments $p<0.0005$ for LTB

Appendix 20. Number and frequencies (%) of ALS IgA responders to ETVAX antigens to the first and second vaccine dose in the different age groups (Cohorts B, C and D)

Age group	CFA/I		CS3		CS5		CS6		LTB	
	Dose 1	Dose 2	Dose 1	Dose 2	Dose 1	Dose 2	Dose 1	Dose 2	Dose 1	Dose 2
24-59 mo N=75	64 (85%)	71 (95%)	70 (93%)	71 (95%)	57 (76%)	57 (76%)	42 (56%)	57 (76%)*	74 (99%)	74 (99%)
12-23 mo N=75	44 (59%)	50 (67%)	43 (57%)	54 (72%)	35 (47%)	39 (52%)	26 (34%)	31 (41%)	63 (84%)	68 (91%)
6-11 mo N=145	35 (24%)	14 (28%)	25 (17%)	47(32%)**	11 (8%)	18(12%)	8(6%)	8 (6%)	58(40%)	95 (66%)***

*$p<0.05$ indicates significant statistical difference between responses to the first and second vaccination in 24-59 months old
**$p<0.01$ for CS3 and ***$p<0.001$ for LTB in infants; differences determined by Fisher's exact test

Appendix 21. Faecal SIgA responses among 6-11 months old infants

Faecal IgA responses (≥2-fold) among infants 6 to 11 months old to ETVAX antigens given placebo or ¼ adult dose with and without dmLT on day 7 and day 28

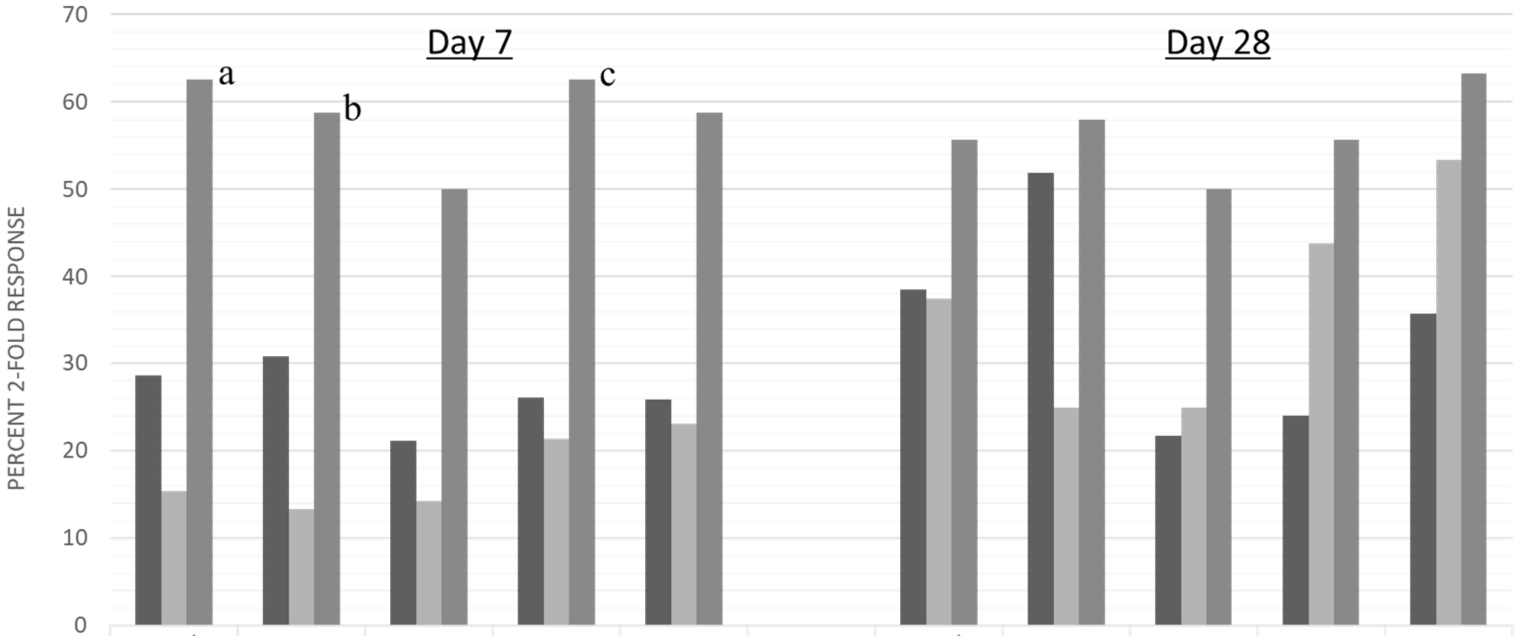

	CFA/I	CS3	CS5	CS6	LTB		CFA/I	CS3	CS5	CS6	LTB
Placebo	28.6	30.8	21.1	26.1	25.9		38.5	51.9	21.7	24.0	35.7
1/4 Dose	15.4	13.3	14.3	21.4	23.1		37.5	25.0	25.0	43.8	53.3
1/4 Dose + 2.5 mcg dmLT	62.5	58.8	50.0	62.5	58.8		55.6	57.9	50.0	55.6	63.2

Comparison of ≥2-fold faecal SIgA responses among 6-11 months old children to ETVAX antigens given placebo or 1/4 adult dose with and without 2.5 µg dmLT on Day 7 (after first dose) and day 28 (14 days after second dose). Statistical differences between vaccine ± dmLT vs. placebo for each vaccine antigen was determined by Fisher's exact test. On Day 7, for CFA/I (a), vaccine + dmLT vs. vaccine alone, $p=0.02$; for CS3 (b), vaccine + dmLT vs. vaccine alone, $p=0.02$; for CS6 (c), vaccine + dmLT vs. vaccine alone, $p=0.03$ and vaccine + dmLT vs. placebo, $p=0.046$; no significant differences between vaccine alone and vaccine + dmLT was noted day 28. Among evaluable subjects who were non-responders to the first dose, after receipt of a second dose of vaccine or vaccine+dmLT, 4-7 and 3-5 more children responded respectively, depending on vaccine antigen.

Appendix 22. Cumulative number and frequencies (%) of 6-11 months old children given a 1/4 dose of vaccine alone or with 2.5 µg of dmLT with ≥2 fold faecal SIgA and/or ALS IgA responses against different numbers of the five primary ETVAX vaccine antigens

Number (%) of children responding to	Placebo n=20	1/4 ETVAX n=30	1/4 ETVAX+ 2.5 µg dmLT n=28
5 antigens	0 (0%)	7 (23%)	12 (43%)
≥4 antigens	3 (15%)	10 (33%)	15 (54%)
≥3 antigens	3 (15%)	15 (50%)	20 (71%)

Responder frequencies were significantly higher by Fisher's exact test in the ETVAX + dmLT versus placebo groups for 5 antigens (p=0.0005), ≥4 antigens (p=0.0078), and ≥3 antigens (p=0.001). Responder frequencies were also significantly different between the vaccine alone vs placebo groups for 5 antigens (p=0.03), but not for ≥4 or ≥3 antigens.

Including dmLT with this fractionated vaccine dose appeared to favor a broader antigenic response than that achieved with the vaccine alone. Mucosal antibody response frequencies were consistently higher in the adjuvanted vaccine group when ≥ 3, ≥ 4 or 5 antigens were considered. A similar positive dmLT effect was observed on the mucosal antibody response in the 12-23 month age group in this study when the antigenic breadth of the responses was considered (data not shown).

Appendix 23. Number and frequencies (%) of infants with ≥4-fold increases in ALS and/or faecal responses against ETVAX antigens in 6-11 month old vaccinees (V) given 1/4 dose alone or with dmLT at any time after immunization

Group (n)	CFA/I	CS3	CS5	CS6	LTB
V + dmLT (n=28)	18 (64%)	13 (46%)	14 (50%)	15 (53%)	20 (71%)
V (n=30)	8 (27%)	10 (33%)	7 (23%)	9 (30%)	23 (77%)
p-value (V vs V+dmLT)	0.008*	ns	ns	ns	ns

* Fisher's exact test; with Holm's Bonferroni adjustment p=0.04 for CFA/I

Note: Vaccine +dmLT compared to vaccine alone appeared to increase frequency of response to each CF with CFA/I responses statistically significant after correcting for repeated measures.

Appendix 24. Study Eligibility Criteria

Inclusion Criteria

- Healthy male or female adults 18-45 years old, inclusive
- General good health as determined by the screening evaluation no greater than 7days before enrollment and vaccination
- Properly informed about the study, able to understand it and sign or thumb print the informed consent form
- Available for the entire period of the study and reachable by study staff throughout the entire follow-up period
- Females of childbearing potential who are willing to take a urine pregnancy test at screening and before the second vaccination. Pregnancy tests must be negative before each vaccination. Females of childbearing potential must agree to use an efficacious hormonal or barrier method of birth control during the study. Abstinence is also acceptable.
- Informed Consent (signature or thumb print provided, with witness signature)

Exclusion criteria

- Presence of any significant known systemic disorder (cardiovascular, pulmonary, hepatic, renal, gastrointestinal, endocrine, immunological, dermatological, neurological, cancer or autoimmune disease) as determined by medical history and/or physical examination which would endanger the participant's health or is likely to result in non-conformance to the protocol.
- History of congenital abdominal disorders, intussusception, abdominal surgery or any other congenital disorder or presence of a significant medical condition that in the opinion of the Investigator precludes participation in the study. Known or suspected impairment of immunological function based on medical history and physical examination. Clinical evidence of active gastrointestinal illness and acute disease at the time of enrollment
- Screening positive with hepatitis B antigen and/or hepatitis C antibodies
- Participation in research involving another investigational product (defined as receipt of investigational product) during the 30 days before planned date of first vaccination or concurrently participating in another clinical study at any time during the study period, in which the participant has been or will be exposed to an investigational or a non-investigational product
- Clinically significant abnormalities in screening hematology or serum chemistry, as determined by the Study Physician
- History of febrile illness within 48 hours prior to vaccination and fever at the time of immunization (fever is defined as a temperature ≥ 37.5°C (99.5°F) on axillary, oral, or tympanic measurement)
- Prior receipt of any cholera (e.g., Dukarol, Shancol) or ETEC vaccine
- Prior receipt of a blood transfusion or blood products, including immunoglobulins
- Evidence of current illicit drug use or drug dependence
- Current use of iron or zinc supplements within the past 7 days; current use of antacids (H2 blockers, omeprazole, OTC agents) or immunosuppressive drug
- Any condition which, in the opinion of the investigator, might jeopardize the safety of study participants or interfere with the evaluation of the study objectives
- Receipt of antimicrobial drugs for any reason within 14 days before vaccination
- History of diarrhea during the 7 days before vaccination (see protocol definition of diarrhea)
- Culture positive for ETEC, *Shigella, V. Cholerae or Salmonella* within 7 days before vaccination.
- Acute disease at the time of enrollment or 3 days prior to enrollment
- History of chronic administration (defined as more than 14 days) of immunosuppressant medications, including corticosteroids.
